# Natural products targeting inflammation-related metabolic disorders: A comprehensive review

**DOI:** 10.1016/j.heliyon.2023.e16919

**Published:** 2023-06-02

**Authors:** Firzan Nainu, Andri Frediansyah, Sukamto S. Mamada, Andi Dian Permana, Mirnawati Salampe, Deepak Chandran, Talha Bin Emran, Jesus Simal-Gandara

**Affiliations:** a Department of Pharmacy, Faculty of Pharmacy, Hasanuddin University, Tamalanrea, Makassar 90245, Indonesia; b Research Center for Food Technology and Processing (PRTPP), National Research and Innovation Agency (BRIN), Yogyakarta 55861, Indonesia; c Department of Pharmaceutical Science and Technology, Faculty of Pharmacy, Hasanuddin University, Tamalanrea, Makassar 90245, Indonesia; d Sekolah Tinggi Ilmu Farmasi Makassar, Makassar 90242, Indonesia; e Department of Veterinary Sciences and Animal Husbandry, Amrita School of Agricultural Sciences, Amrita Vishwa Vidyapeetham University, Coimbatore 642109, India; f Department of Pathology and Laboratory Medicine, Warren Alpert Medical School & Legorreta Cancer Center, Brown University, Providence, RI 02912, USA; g Department of Pharmacy, Faculty of Allied Health Sciences, Daffodil International University, Dhaka 1207, Bangladesh; h Department of Pharmacy, BGC Trust University Bangladesh, Chittagong 4381, Bangladesh; i Universidade de Vigo, Nutrition and Bromatology Group, Analytical Chemistry and Food Science Department, Faculty of Science, E32004 Ourense, Spain

## Abstract

Currently, the incidence of metabolic disorders is increasing, setting a challenge to global health. With major advancement in the diagnostic tools and clinical procedures, much has been known in the etiology of metabolic disorders and their corresponding pathophysiologies. In addition, the use of *in vitro* and *in vivo* experimental models prior to clinical studies has promoted numerous biomedical breakthroughs, including in the discovery and development of drug candidates to treat metabolic disorders. Indeed, chemicals isolated from natural products have been extensively studied as prospective drug candidates to manage diabetes, obesity, heart-related diseases, and cancer, partly due to their antioxidant and anti-inflammatory properties. Continuous efforts have been made in parallel to improve their bioactivity and bioavailability using selected drug delivery approaches. Here, we provide insights on recent progress in the role of inflammatory-mediated responses on the initiation of metabolic disorders, with particular reference to diabetes mellitus, obesity, heart-related diseases, and cancer. In addition, we discussed the prospective role of natural products in the management of diabetes, obesity, heart-related diseases, and cancers and provide lists of potential biological targets for high throughput screening in drug discovery and development. Lastly, we discussed findings observed in the preclinical and clinical studies prior to identifying suitable approaches on the phytochemical drug delivery systems that are potential to be used in the treatment of metabolic disorders.

## 1 Introduction

All life forms, including humans, require a highly orchestrated process, is termed as metabolism, to break down the ingested foods to become their simpler elements [254]. In eukaryotes, this process is essential to provide energy required for a species to develop and live. Failure to do so will negatively affect the species survival [322]. Unfortunately, we have witnessed an increasing trend of metabolism-related problems, simplified as metabolic disorders in recent years. People with metabolic disorders may have different characteristics compared to the ones with normal metabolism. Such discrepancy may occur as a result of certain pathological condition that leads to distinct phenotypes [173, 254].

At present, the most predominant metabolic disorders are diabetes mellitus, obesity, heart-related diseases, and cancer [254]. Although much has been known regarding the etiology and pharmacological management of these metabolic disorders, the mechanistic basis is complex and remains to be fully elucidated. Nevertheless, chronic inflammation appears to be one of the key players in the initiation, progression, and transition of the abovementioned metabolic disorders [103,129,225,269]. Stimulation of various pro-inflammatory cytokines in response to the release of endogenous yet danger-associated ligands have been observed to occur in most of the, if not all, metabolic disorders-related condition [129, 131, 269].

Growing evidence indicates that natural products and their bioactive compounds, particularly phytochemicals, can provide various benefits to the human health. Indeed, one of the most focused natural products research areas is the potential application of phytochemicals to treat diabetes, obesity, cardiovascular-related problems, and different types of cancers [254], possibly by targeting the oxidative stress-related pathways and regulatory network of inflammatory process [16,20]. In this review, we discussed a current understanding on the pathophysiology of diabetes, obesity, heart-related diseases, and cancers in correlation with inflammation-mediated induction of metabolic disorders. Furthermore, we later provide a brief and concise discussion on the prospective role of natural products in the management of the diabetes, obesity, heart-related diseases, and cancers by listing the potential biological targets for the phytochemicals and findings observed in the preclinical and clinical studies prior to describing current approaches on the phytochemical drug delivery systems that have been used in the treatment of metabolic disorders.

### 1.1 Inflammation-mediated induction of metabolic disorders

Survival mechanisms like as metabolic and immunological systems are crucial. Many mechanisms involved in metabolism and immunity, as well as systems that detect nutrients and pathogens, have been conserved across species. Therefore, metabolic control and immunological response are intricately linked, with the health of one depending on the other. The malfunction of this interface has been linked to a variety of chronic metabolic illnesses, including obesity, type 2 diabetes, and cardiovascular disease, and hence can be thought of as a central homeostatic mechanism [130, 158, 164, 225]. As a group, these illnesses pose the greatest danger to the health and well-being of people around the world today.

#### 1.1.1 Implications for the metabolism-inflammation link

The maintenance of metabolic balance depends on insulin, the primary anabolic hormone in animals. Cellular substrates of insulin, including the insulin receptor substrate (IRs) family of proteins, are tyrosine phosphorylated when insulin binds to their receptor. Although changes like serine phosphorylation, regulated by intracellular regulatory pathways, are essential for mediating many of insulin's metabolic actions, they are suppressed under conditions of stress and inflammation [240,289]. People who are overweight, insulin resistant, or have type 2 diabetes also showed this inhibition. Immune mediators, such as cytokines like tumor necrosis factor (TNF)-α, may play a vital regulatory role in systemic glucose homeostasis, as they can initiate the alterations that reduce insulin's efficacy [201]. Insulin signaling is a highly conserved and dominant metabolic route in nutrition and energy homeostasis, and it has been shown that inflammation can contribute to metabolic dysregulation at multiple levels [204,225].

Exploring the connections between immune responses and metabolic regulation has benefited greatly from the discovery platform provided by the identification of the relationship between inflammation and insulin signaling [14, 214]. Nutrients, such as circulating lipids, directly stimulate many of the inflammatory signaling pathways that impede insulin-receptor signaling [164]. Organelle stress caused by nutritional excess and processing errors leads to metabolic stress, which in turn induces further inflammatory pathways. The serine phosphorylation of IRs1 in both circumstances results in the disruption of the insulin signaling system and different metabolic responses due to the activation of kinases such as JUN N-terminal kinase (JNK; also known as maPK8) and Iκb kinase-β (IKKβ). Immune signaling pathways can also activate extracellular-signal-regulated kinase (ERK), ribosomal protein S6 kinase (S6K; also known as RPS6KB1), mammalian target of rapamycin (mTOR; also known as FRAP1), protein kinase C, and glycogen synthase kinase 3, all of which can disrupt the insulin signaling pathway [90,104,289]. It is likely that changes in metabolic responses will be connected to a wide variety of immunological signaling pathways and proteins. Moreover, metabolic signaling pathways might influence the immunological response. The inflammatory response can be dampened, for instance, by turning on nuclear receptors such as peroxisome proliferator-activated receptors (PPARs) and liver X receptors (LXRs). Several other metabolic hormones, including leptin, resistin, and adiponectin, have immunological functions as well [158, 238].

#### 1.1.2 How does inflammation trigger metabolic dysfunction?

• Pattern recognition receptors (PRRs) as metabolic sensors

It is well known that PRRs in the innate immune system may detect foreign molecules (pathogen-associated chemical patterns) and launch a defense response. But it is now known that the ability of PRRs to identify endogenous ligands generated in the obese state is a trigger in obesity-associated inflammation [179]. The Toll-like receptor 4 (TLR4) is the most studied PRR because it responds to free fatty acids (FAs) by producing inflammatory signals and activating the nuclear factor kappa B (NF-κB). Obesity-induced inflammatory activation is prevented in TLR4-deficient mice, and these mice also show resistance to insulin infusion-induced fat gain [152]. Although leukocytes play a role in mediating this effect, there is strong evidence that TLR4 activation in non-hematopoietic cells has direct consequences on the metabolic phenotype [14]. Nearly all members of the TLR family are expressed in adipose tissue, and TLR2-knockout mice are protected from high-fat DIO and insulin resistance, indicating a broad function for TLRs in obesity and its associated morbidities. Mice lacking TLR5 exhibit obesity and insulin resistance due to changes in their gut microbiome, demonstrating that TLRs monitor and control gut microorganisms in a way that contributes to metabolism in addition to FAs [179,238].

Obesity-induced signals are also detected by the Nod-like receptor (NLR) family of PRRs. Leukocytes are directed toward stimuli that activate NLRs in order to limit tissue damage. NLRs are triggered by danger signals from stressed or dying cells. When NLRs are activated, caspase-1 is activated to produce IL-1β and IL-18 in macrophages. When glucose levels remain high for an extended period of time, cells in the pancreas begin to die. Diet-induced obesity (DIO) also induces caspase-1 and IL-1β in adipose tissue, and NLRP3- and caspase-1-deficient mice are resistant to DIO-induced inflammation [130, 158]. Mice lacking NLRP3 exhibit reduced M1 and increased M2 gene expression without quantitative changes in adipose tissue macrophages (ATMs), suggesting that changes in the M1 activation of ATMs underlie this protective effect. Numerous mechanisms may contribute to meta-inflammation, if PRRs can serve as universal dual sensors of pathogenic and endogenous signals pertinent to obesity [225].

• IKKβ and NF-κB

Multiple pathways, some of which may or may not involve the adaptor protein MyD88, are involved in transmitting intracellular signals that are triggered by TLR activation. MyD88−/− mice are more prone to insulin resistance with DIO, although the significance of MyD88-dependent signaling in other metabolic organs remains elusive [158]. When a person is obese, the activation of IKKβ happens downstream of MyD88 and plays a crucial role in inflammation throughout the body, particularly in the liver, myeloid cells, and hypothalamus. Salicylate, an IKKβ inhibitor, is under clinical trials for the treatment of type 2 diabetes, and its insulin-sensitizing effect is likely due to this inhibitor's broad spectrum of activity [54,68]. TLR/IKKβ signals are ultimately translated into NF-κβ-dependent activation of inflammatory gene transcription. DIO induces NF–B expression primarily in adipose tissue and atrial myocytes, as seen by *in vivo* imaging. One NF-κβ-sensitive gene activated by high-fat diet is *Ikke*, a protein kinase that appears to play a role in regulating body weight and insulin resistance by inhibiting thermogenesis. There are still questions about how to tell the difference between the metabolic effects of acute and chronic NF-κβ activation, and this highlights the significance of temporal management of NF-κβ activation. Acute exercise in lean individuals, for instance, causes a temporary release of proinflammatory cytokines like IL-6 from muscle NF-κβ [267].

• Role of ceramides and intracellular lipids in inflammation and metabolic processes

There are other implications of TLR4 activation beyond NF-κB activation. The equilibrium between intracellular lipid species like ceramides and sphingolipids may play an important role in both metabolism and inflammation [240]. Saturated FAs propensity to promote insulin resistance is prevented by ceramide synthesis inhibition. TLR4 is required for lipopolysaccharide (LPS) and saturated FA-induced ceramide formation in numerous metabolic organs, including the brain and muscle, where it can block insulin signaling via the Akt pathway. Salicylates lower ceramide levels in the liver, muscle, and hypothalamus, indicating that IKKβ is required for TLR4-mediated ceramide synthesis in metabolic organs [152].

Adiponectin, an adipokine, has been known for a long time to have beneficial effects on a variety of cell types, including increasing insulin sensitivity and decreasing the activity of proinflammatory pathways. Because adiponectin increases ceramidase activity and alters the ratio of ceramides to sphingosine-1-phosphate, control of ceramides may be a mechanism by which adiponectin exerts its effects [130]. Protecting against cardiomyocyte and cell apoptosis suggests that adiponectin's effect on cellular ceramide concentration is significant for numerous organs. It is possible that adiponectin receptor-associated ceramidase activity is not the only factor at play [14]. Adiponectin infusion increased insulin sensitivity in hepatocytes via IRS2 activation, as discovered by Ref. [19]; however this effect was not cell autonomous [19]. This insulin-sensitizing effect was unexpectedly caused by the activation of IL-6 by adiponectin in macrophages, and it occurred substantially independently of the adiponectin receptors R1 and R2.

• JNK and stress

Through upstream pathways shared by IKKβ/NF-κB in response to stress signals including fatty acids (FAs), insulin, hyperglycemia, and inflammatory cytokines, obesity also activates JNK in insulin-responsive tissues. In comparison to other components of inflammatory signaling, the unique role played by JNK in hematopoietic and non-hematopoietic cells in obesity is well characterized [14, 238]. Even though both JNK1 and JNK2 isoforms play a part in metabolic control, JNK1 has a more significant role in DIO protection. Body weight and energy expenditure are regulated by JNK1's actions in nonhematopoietic cells. Inactivating JNK1 in the hypothalamus protects mice against DIO and mimics the lower body weight phenotype found in JNK1-deficient animals. The IKK pathway is also involved in the regulation of hypothalamic signals. Although inhibiting JNK1 in hematopoietic cells does not affect adiposity, it is sufficient to reduce the inflammation brought on by obesity, which has positive metabolic consequences [130].

Activation of JNK1 and IKKβ/NF-kB appear to be tightly linked to ER stress and the downstream activation of the molecular pathways directing the unfolded protein response in a variety of metabolic organs (e.g., hypothalamus and adipose tissue). Obesity is characterized by widespread activation of ER stress signaling components and cascades (ATF6, PERK, IRE-1), and therapeutic suppression of ER stress can correct metabolic abnormalities [142,225]. At the crossroads of ER stress and nutrition is the PRR represented by the double-stranded RNA-dependent protein kinase, which in turn translates these signals into an inflammatory response via the coiled-coil domain of the JNK. More research is required to determine the extent to which ER stress is present in different acute and chronic stress scenarios and how its mechanism coincides with its role in the pathogenesis of atherosclerosis and foam cell biology [246].

### 1.2 Pathophysiology of metabolic disorders: what we have known so far

#### 1.2.1 Diabetes mellitus

The pathophysiology of diabetes mellitus is closely associated with two essential factors, i.e., insulin levels and the body's ability to utilize this hormone. Insulin is the key determinant responsible for assisting the entry of blood glucose into the cells to be metabolized for yielding energy. Therefore, any conditions affecting the physiological roles of insulin will result in disturbances of glucose levels.

**Fig. 1:**
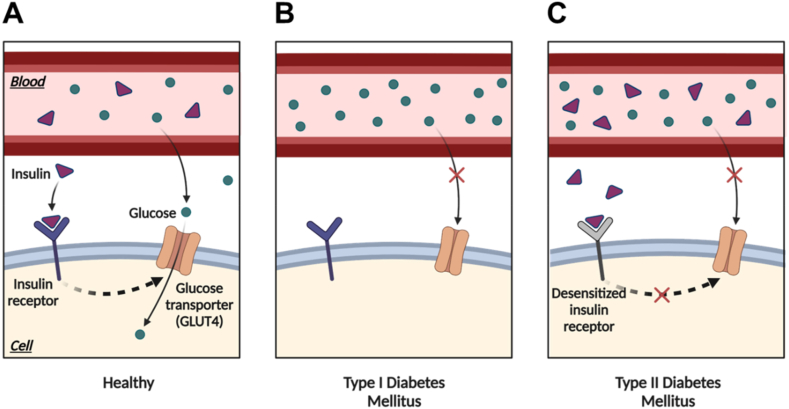
Physiological regulation of glucose in a healthy cell (A) and pathophysiological differences between the T1 (B) and T2 (C) DM. In healthy cells (A), glucose is transported into the cells using GLUT4 in the presence of insulin. When blood glucose levels rise, insulin is released and binds to its receptor on the cell surface. This activates a series of events leading to translocation of GLUT4 transporters to the cell surface. With GLUT4 transporters now present in the membrane, glucose can bind to them and enter the cell. However, in the T1DM (B), the lack of insulin production or absence of insulin prevents the proper translocation of GLUT4 transporters to the cell surface. Without sufficient insulin, GLUT4 remains trapped inside intracellular vesicles, impairing glucose uptake into cells. This leads to elevated blood glucose levels. In T2DM, insulin resistance can disrupt the transport of glucose inside cells using GLUT4. Insulin resistance reduces the effectiveness of insulin in promoting glucose uptake, thus, decreases glucose uptake into cells, leading to elevated blood glucose levels. GLUT4, glucose transporter 4; T1DM, type 1 diabetes mellitus; T2DM, type 2 diabetes mellitus.

Several types of diabetes mellitus have been introduced; however, the type 1 (T1) and type 2 (T2) diabetes mellitus (DM) seem to be the most recognized types of diabetes. Although both types show different pathogenesis mechanisms, the inability of the insulin to be utilized by the cell to facilitate the entry of the glucose is the main pathophysiological event in both T1DM and T2DM (see Fig. 1).

• Type 1 diabetes mellitus (T1DM)

A condition where the function of pancreatic beta cells is disturbed leading to their inability to produce insulin anymore could result in the emergence of T1DM. It is now concluded that the failure of the pancreatic cells to produce insulin is closely linked to a condition called autoimmune disease [132]. Instead of protecting the body from foreign substances, the immune system attacks the other systems, tissues, or cells in the autoimmune disease, including insulin-producing pancreatic beta-cells. When the latter is attacked, their function to produce a proper insulin level is damaged. Consequently, blood glucose level increases significantly leading to the emergence of hyperglycemia manifestation.

Although many cornerstones have been achieved in recent years on the pathophysiological aspects of T1DM, no clear answer could explain the autoimmune condition of this type of diabetes. However, several things related to the involvement of the immune system in the emergence of T1DM have become more evident and are revealed. First, it was found more than three decades ago that the expression of a molecule called human leukocyte antigen (HLA) was relatively higher in diabetic patients [25]. As this molecule is pivotal in regulating the immune response by encoding various related proteins involved in the antigen presentation, any condition altering the expression and function of this molecule may lead to the loss of self-tolerance mechanisms [308].

Secondly, the role of humoral and cellular immunity is significant in the pathogenesis of type 1 diabetes mellitus. As inflammation is inherently involved in the course of the disease, the excessive action of the immune cells, including T lymphocytes and B lymphocytes, is unavoidable [49]. The link between the latter cells and T1DM was established almost 50 years ago when Bottazzo and co-workers demonstrated the presence of autoantibodies for pancreatic islet cells in patients suffering from type 1 diabetes mellitus [31]. More recently, Wilcox and colleagues reported that T lymphocytes also played a significant role in T1DM as these immune cells were the dominant immune cells found in pancreas samples collected from 29 diabetic patients after doing post-mortem analysis [291].

Like other autoimmune diseases, the emergence and progression of T1DM are linked to the time of development. At this point, Eisenbarth published a paper proposing the putative pathological stages of T1DM [72]. In this concept, three previous stages would be experienced by a patient before type 1 diabetes mellitus diagnosis is established. In the first stage, when the mass and function of pancreatic beta cells are still normal, some triggering factors play important roles in activating the self-targeting immune pathway that could attack the beta cells. In stage 2, autoimmunity has been detected as autoantibodies against the beta cells could be observed in this stage. However, at this stage, the individual still has normal blood glucose and insulin levels indicating the reduced mass of the beta cells in the second stage is still sufficient to supply the need for insulin. As time goes by, the next stage is characterized by the significant reduction of the mass and function of the beta cells, leading to hyperglycemia. In the final stage, when the diagnosis of T1DM is established, the lack of beta cell mass is observed resulting in the total dysfunctionality of the cell to produce insulin [72,308].

• Type 2 diabetes mellitus (T2DM)

Unlike T1DM, severe hyperglycemia in the T2DM patient is not primarily caused by the destruction of beta cells. Conversely, this pathogenic condition is induced by the failure of the peripheral tissues and cells to utilize insulin leading to their inability to uptake blood glucose. This condition is known as insulin resistance. As a consequence, hyperglycemia occurs even though the insulin circulating in the blood is at the physiological level. Following this condition, the vicious cycle occurs when the beta cells keep producing insulin because they constantly receive "information" that the circulated glucose level still exceeds the normal level [308]. If this event keeps happening, when the diagnosis of T2DM is established, the beta cells have been in a failed condition to secrete insulin.

Several factors have been proposed to play important roles in regulating the action of the beta cells to produce insulin. One of the relatively new concepts is the role of gut-related hormones (also known as incretins). It has been known that two gut hormones act as a messenger to stimulate insulin secretion after ingesting glucose. However, this mechanism is not fully activated when the supply of glucose is given intravenously. Those hormones are glucagon-like peptide-1 (GLP-1) and gastric inhibitory polypeptide (GIP) [137]. To maintain blood glucose levels after food consumption, both incretins stimulate insulin production, while only GLP-1 shows the ability to decrease glucagon secretion. It has been demonstrated that in T2DM, the secretion of the incretins, especially GLP-1, is lowered significantly leading to the failure to induce insulin production after food ingestion [66]. Inversely, glucagon level increases facilitating the conversion of glycogen to glucose. Collectively, these events result in the elevation of blood glucose levels.

The role of the kidney in regulating blood glucose levels has also been established. This role is closely linked to kidney function in the reabsorption of glucose in the tubules after passing the filtration in the glomerulus. Approximately 90% glucose reabsorption occurs in the proximal tubules via the action of the sodium-glucose cotransporter 2 (SGLT2) membrane transporter, while the rest is reabsorbed in the descending tubule in the loop of Henle through SGLT1 [92,308]. As an important note, the reabsorption process keeps taking place until the maximum reabsorption capacity is achieved at 200 mg/dL [1]. It has been noticed that this capacity increases in patients suffering from T2DM. As a result, the event of hyperglycemia is exacerbated.

The exact mechanism by which insulin resistance occurs is still blurry. However, the link between insulin resistance and fat accumulation as well as obesity is more explicit. It has been demonstrated that the liver and muscles play a significant role in the emergence of insulin resistance. This role is putatively linked to their capacity to store excessive fats in the body [57,308]. The excessive accumulation of fat in several sites, particularly liver and muscle, has been accepted as one of the determinants involved in initiating reduced insulin sensitivity. Many factors take part in creating the accumulation of fat in those tissues or organs. Still, it is evident that the excessive supply of calories not followed by the proper physical activity, often observed in the state of obesity, plays a significant role. Specifically, fat accumulation in beta cells could destroy their function so that they cannot produce insulin at the physiological level and eventually fail to maintain the level of blood glucose [28,231].

Finally, the genetic aspects also play a role in the pathophysiology of T2DM. Although some sources have mentioned that T2DM does not have a strong pattern of inheritance, some genetical aspects should be observed carefully as family history and genetic predisposition have been known as one of the risk factors of T2DM [82, 299].

#### 1.2.2 Obesity

As various factors, including environmental, social, behavioral, physiological, medical, and genetic factors, contribute to the emergence and persistence of obesity, the pathogenesis of this condition is complex [80]. In terms of environmental factors, a number of lifestyles are modified following the success of controlling infectious diseases that were the main cause of death in the previous centuries followed by multiple technological achievements. For example, the installation of various transportation modes and easy access to electronic and portable devices have minimized physical activities. This condition is exacerbated by easy access to high-calorie foods.

Genetic factors also contribute to the pathogenesis of obesity. Surprisingly, the heritability of body mass index ranges from 40 to 70% [34]. Several monogenic mutations or changes linked to the pathogenesis of obesity have been identified. Of those, deficiency of the leptin and melanocortin-4 receptors attracts more interest. These receptors regulate human energy homeostasis [99,270]. Several studies have demonstrated that in obesity, a deficiency of these proteins is often detected [202, 277].

Genetic and environmental factors play essential roles in influencing various physiological systems responsible for energy homeostasis. One of those systems is the nervous system. Guarino and colleagues proposed the importance of the autonomic nervous system in the pathophysiology of obesity [86]. Specifically, this group underlined the increased sympathetic nervous system activity in obese individuals [86].

**Fig. 2:**
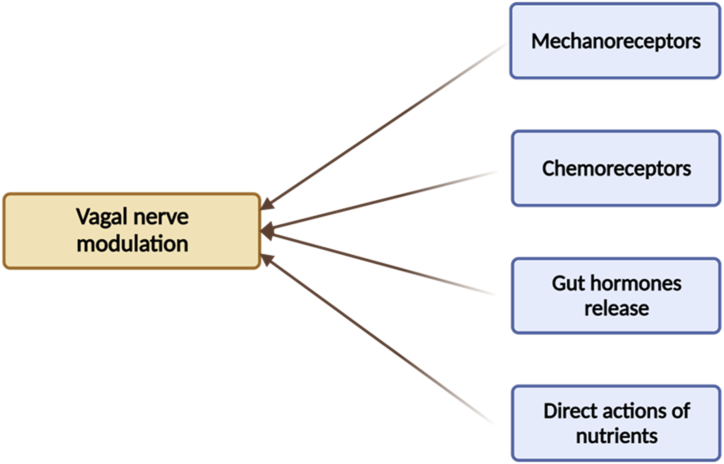
The role of vagal nerve is essential for regulating energy-balance ratio. The action of this nerve is affected by the information received from the mechanical stimulation, gut hormones release, chemoreceptors activation, and direct actions of some nutritive compounds.

Furthermore, the vagal nerve is also linked to the pathogenesis of obesity as this nerve is the main link bridging the brain and the gut for the modulation of satiety [24]. This nerve receives information from the gut after ingesting process via several ways, i.e., mechanical stimulation, gut hormones release, chemoreceptors activation, and direct actions of some nutritive compounds (Fig. 2) [37, 86, 110]. While the first way is stimulated by gastric distension after feeding, the second way is mediated by various gut hormones. To date, a number of gut hormones have been identified, including cholecystokinin, peptide YY (PYY), pancreatic polypeptide (PP), glucagon-like peptide-1 (GLP-1), ghrelin, insulin, and leptin [86]. Although these hormones play different functions, the final aim is to regulate food intake and gastric emptying.

Upon receiving the information from the peripheral receptors, vagal nerve projects the information to the complex of area postrema and nucleus of the solitary tract in the brainstem where the information is processed to be further projected to the dorsal motor nucleus [86]. The modulation of this pathway may cause several events associated with the gastric emptying control, absorption rate, and changes in the secretion of the gut hormones [24, 76, 86]. Given the essential role of the vagal nerve, any conditions that can cause disturbances in the action of this nerve in receiving information from the gut could lead to energy-balance dysregulation.

#### 1.2.3 Heart-related diseases

As there are many types of heart diseases with their characteristics and due to space limitations, we do not provide pathophysiological aspects of each type of heart-related disease in this part. We select coronary artery disease (CAD) as the representative.

**Fig. 3:**
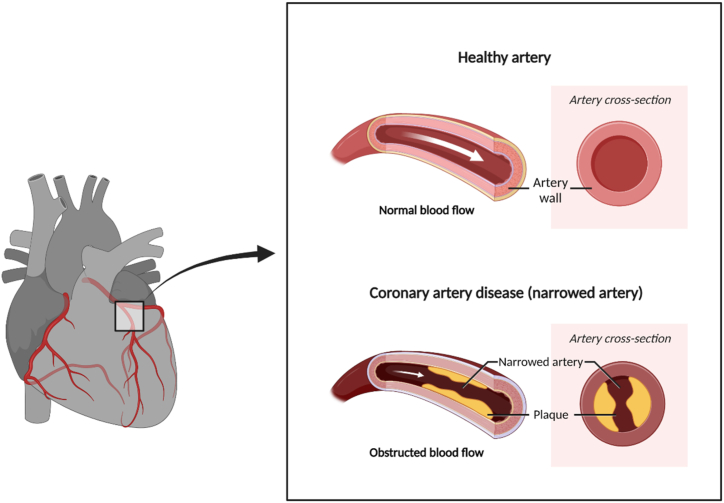
The pathogenesis of coronary artery disease. As the coronary artery is blocked, the blood supply needed by the heart would not be sufficient leading to a condition where the insufficiency of oxygen and nutrients occurs.

As its name suggests, CAD occurs when there is an obstruction in the coronary arteries. These vessels supply blood to the heart, ensuring the organ gets sufficient oxygen and nutrients. Once these arteries are blocked, the heart will not work correctly as it has no adequate energy to run its function (Fig. 3).

Although several causes of artery blockage have been listed, atherosclerosis becomes the leading cause of blocking the blood flow in the arteries. Atherosclerosis could be initiated when a low-grade inflammation is detected in the inner layer of the medium-sized arteries, including the coronary [11]. Several risk factors, including hypertension, high blood cholesterol level, diabetes mellitus, and genetics, are known to worsen this condition [11]. Although the pathogenic process in atherosclerosis is considered to be slow, this progression results in the thickening of the intima layer of the coronary occurring gradually [11]. Over time, this pathogenic event is followed by the narrowing process of the artery lumen. However, several factors can shift the slow progression of atherosclerosis to rapid atherosclerotic progression. Those factors are the formation of plaque hemorrhage and the non-occlusive thrombus in the intraluminal area [11].

• Formation of plaque hemorrhage

The thickening of the intima layer of the coronary during atherosclerosis disturbs its blood supply. Therefore, a compensation mechanism is activated where the vessels that originally nourish the outer layer of the arteries grow and supply the intima layer with nutrients and oxygen [85]. Unfortunately, these growing vessels possess thin walls and weak endothelial integrity. Therefore, these vessels are vulnerable to suffer from rupture. Once the rupture occurs, the blood cells experience deposition and subsequently enlarge the plaque size. This condition is exacerbated by the fact that the red cell membrane contains high lipids, making the plaque formed rich in lipids and vulnerable to inflammation [144].

Intriguingly, the arterial lumen does not narrow easily in the initial phase of plaque formation. Several compensations and remodeling mechanisms help the affected artery maintain its lumen diameter. However, when the plaque volume approaches 40%, these mechanisms cannot compensate for the pathological effects that emerge from the formed plaque [85].

• Formation of thrombus

Several major contents of an atherosclerotic plaque have been identified as inflammatory cells, including macrophage foam cells, debris from dead cells, and cholesterol in various forms [151]. These core contents of plaque are formed under the fibrous cap mainly composed of collagen, elastin, and smooth muscle cell. As the luminal side of the cap is lined by only a single layer of endothelial cells, the atherosclerotic plaque is vulnerable to experiencing tears [29]. This vulnerability gets more prominent in the presence of the inflammatory cells-derived foam cells responsible for weakening and thinning the fibrous cap [11].

Once the fibrous cap tears, the plaque core is exposed to the circulated blood, forming the coronary thrombus. The formed thrombus does not necessarily follow the flowing blood direction as other events might also occur, e.g., the thrombus is lysed and incorporated again into the arterial wall. This process is responsible for the further narrowing of the arterial lumen. Following the tear of the fibrous cap, the thrombus can also experience further growth and progress so that a total coronary lumen occlusion could occur [11]. Several factors determine which mechanism would be followed by the formed thrombus, e.g., the size, the volume, and the contents of the plaque [249].

#### 1.2.4 Cancer

**Fig. 4:**
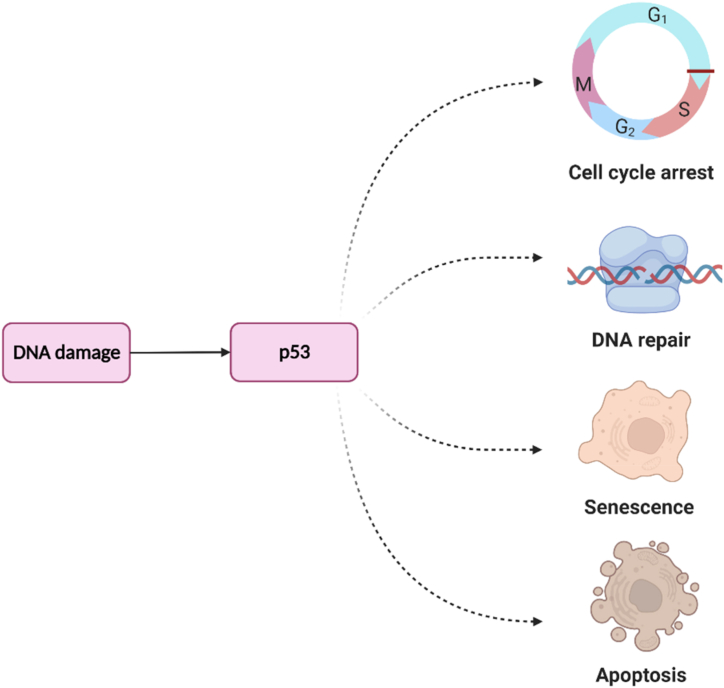
Main mechanisms by which p53 deals with DNA damage. p53, a tumor suppressor protein, employs multiple mechanisms to address DNA damage. It halts the cell cycle, activates DNA repair, promotes cellular senescence, and induces apoptosis. These processes collectively maintain genomic stability, prevent the propagation of mutations, and inhibit the development and progression of cancer.

The pathogenesis of cancer is closely linked to DNA damage. As our cells are continuously exposed to various stresses that could lead to damage of DNA, several mechanisms have been developed by our body to mitigate the affected DNA, i.e., cell-cycle arrest, DNA repair mechanism, cellular senescence, and induction of apoptosis (Fig. 4) [156]. These mechanisms are strictly regulated by the p53 family (p53, p63, and p73) appointed as the “guardian of the genome” [199]. At this point, any conditions that destruct the functionalities of the p53 family could lead to the emergence of cancerous events.

More than half of human cancers are linked to missense mutations in the p53 family [186,199]. Of several sites of mutation, the DNA-binding domain (DBD) site seems to be the most vulnerable site for mutation in the family of p53 [199]. In normal conditions, the expression of p53 must be maintained at a very low level. However, when a particular stressor attacks a cell, p53 immediately upregulates the expression of the murine/human double minute 2 (MDM2). Interestingly, MDM2 also has an activity to exert a negative feedback mechanism for p53 so that the expression of p53 is prevented from being higher [156].

As stated above, the mitigation of the damaged DNA carried out by the p53 family consists of apoptosis, cell-cycle arrest, and senescence mechanisms. In the former mechanism, p53 could initiate apoptosis through extrinsic or intrinsic pathways. While the action of the death receptors mediates the former pathway, the latter pathway is associated with the release of cytochrome *c* to the cytosolic region of mitochondria [219]. In the final stage of both pathways, caspase-3 seems to play a major role in executing the apoptotic events [87,236].

Another strategy for mitigating the damaged DNA is the activation of the cell-cycle arrest. This strategy is essential for evaluating the ongoing process and repairing the damage during the cell cycle. To facilitate and activate the cell cycle process, the role of the cyclin-dependent kinases (CDKs) family is crucial, while the inhibition of this protein family is linked to the termination of cellular duplication which is beneficial for preventing the division of cancer cells. The p53 family regulates the CDKs as p53 could induce the activation of the p21 protein which is responsible for inhibiting the CDKs [156, 199]. Finally, cellular senescence could also be activated by p53 through its action on some genes, e.g., p21, p16-Rb, and BTG2 [148, 199]. This mechanism is pivotal, particularly in diminishing the progression and spread of cancer cells.

Some other mechanisms are also linked to the action of p53 in protecting the cells from the attack of cancerous cells. Some of them are associated with its ability to prevent several events, e.g., cancer migration to other tissues, angiogenesis, oxidative stress, and drug resistance. In addition, p53 could also induce autophagy and promote genome stabilization [26, 98, 199, 304].

### 1.3 Prospective biological targets for natural products to manage metabolic disorders

To date, the potencies of natural products on tackling metabolic syndromes have been widely explored. Several reasons underlie the efforts carried out for seeking new candidates for those pathologic conditions. Those reasons are from the ineffective existing drugs, the adverse side effects showed by the existing drugs, drug-interaction issues, dosage used for therapy, to the unaffordable price. It is assumed that those drawbacks could be tackled by new drugs developed from natural products. However, excessive exploration on the natural products is linked to the harmful impact on the nature. Therefore, although the nature stores the priceless entities for being developed as a drug, the exploration of the nature should be carried out wisely.

Here we listed several natural compounds that have been reported to show potencies to alleviate diabetes mellitus and obesity (Table 1), heart-related diseases (Table 2), and cancer (Table 3). We equipped the lists with the sources from which the compounds are extracted, the putative mechanism(s) of action of each compound, the models used during the experiments, and the key findings of the studies that we cited. In addition, we also provide a list of natural compounds with protective effect against diabetes mellitus, obesity, heart-related diseases, and cancer by specifically modulating excessive effects of proinflammatory cytokines (Table 4).

**Table 1:** Prospective biological targets for natural products to manage diabetes mellitus and obesity.

No	Source(s)	Compound or extract(s)	Mechanism of action(s)	Experimental model(s)	Key findings	Refs
1	*Psidium guajava*	Triterpenoid (Corosolic acid)	α-glucosidase inhibitor	An *in vitro* assay of α-glucosidase inhibition	Corosolic acid derived from *P. guajava* extract exhibited the best inhibition of α-glucosidase among nine triterpenoids isolated with IC_50_ value of this compound was 1.33 μg/mL. However, the result showed that the extract of *P. guajava* leaves was more effective than the individual of its compounds.	[42]
		The ethyl acetate fraction of leaves extract	Modulates advanced glycation end products, Serum fructosamine, and fasting blood glucose levels	An *in vivo* study using rats induced by streptozotocin to provide diabetic myocardium	• Advanced glycation end products of diabetic rats treated by the fraction were significantly decreased to near-normal levels. Similarly, the administration of the fraction in all diabetic groups significantly reduced the fasting blood glucose levels. •To evaluate the beneficial effects of *P. guajava* leaves on diabetic myocardium, the heart-to-body-weight ratio decreased by 10% in diabetic rats treated with a fraction dosage of 25 mg/kg body weight/day.	[247]
		Ethanol extracts from leaves and bark	α-glucosidase, α-amylase inhibitor; stimulate glucose uptake in muscle; inhibit liver glucose production and triglyceride accumulation in adipocytes	An *in vitro* study using the cell lines (H4IIE, C2C12, and 3T3-L1)	•Ethanolic extracts of *P. guajava* leaves and bark vigorously inhibited α-glucosidase with IC_50_ values of 1.0 ± 0.3 and 0.5 ± 0.01 μg/mL, respectively. •In the α-amylase inhibition assay, the ethanolic extract of *P. guajava* bark showed an IC_50_ value of 10.6 ± 0.4 μg/mL. In contrast, the leaves extract gave IC50 value in the range of the three highest concentrations up to 1000 μg/mL. •In addition, *P. guajava* leaves extract at 50 μg/mL showed the same level of glucose uptake as metformin at 400 μM and insulin at 100 nM.	[22]
		Ethanol extract from leaves	Inhibits glucose absorption	An *in vivo* study with an alloxan diabetes test method and oral glucose tolerance test in rats	Administration of extract at 1,300 mg/kg BW each day for 14 days lowered blood glucose levels, indicating that ethanol extracts inhibited blood glucose absorption by promoting its antidiabetic agent as an α-glucosidase inhibitor.	[180]
		Methanol extract from leaves	Increases glucose uptake	An *in vitro* study using glucose uptake in 3T3-L1 cells	The glucose uptake significantly increased by approximately 52% at a concentration of 100 μg/mL of extract.	[45]
			Adipogenesis and lipolysis	An *in vitro* study using adipogenesis assay, and lipolysis assay in 3T3-L1 cells	Guajava leaves extract (GLE) decreased lipid accumulation during adipocyte differentiation. Lipid content could be reduced by approximately 88%, and the glucose uptake significantly increased by approximately 52% at a concentration of 100 μg/mL GLE.	
2	*Ficus tikoua* Bur.	n-butanol fraction (NBF) of Ethanol extract	• Stimulates glucose uptake via P13K/AKT and AMPK pathway • α-glucosidase inhibitor	An *In vitro* study using 3T3-L1 cells and *in vivo* experimental models in mice	•NBF was potent as an α-glucosidase inhibitor with an IC50 value of 0.89 ± 0.04 μg/mL. In addition, NBF exerted its effects by increasing glucose uptake in 3T3-L1 adipocytes in a dose-dependent manner. •Blood glucose levels in Oral Glucose Tolerance Test (OGTT) and Insulin Tolerance Test (ITT), as well as HbA1c, were significantly lower than in model groups. •The upregulation of p-PI3K and p-Akt in 3T3-L1 adipocytes might mediate the possible mechanism of NBF in increasing glucose uptake.	[282]
3	*Ganoderma resinaceum*	Triterpenoid lactones	α-glucosidase inhibitor	An *in vitro* α-glucosidase inhibitory assay	Compounds 1 and 2 were more potent α-glucosidase inhibitors than acarbose, with IC50 values of 0.75 ± 0.018 mM and 1.64 ± 0.022 mM, respectively.	[44]
4	*Cyclocarya paliurus*	Triterpenoid glycosides isolated from leaves ethanol extract	Increase glucose uptake via AMPK/p38 pathways	An *in vitro* study in 3T3-L1 adipocytes and C2C12 myotubes	Compound 1 significantly enhanced insulin-stimulated glucose uptake in 3T3-L1 adipocytes and C2C12 myotubes. The promising mechanisms of compound 1 in enhancing glucose uptake in cells are upregulating the AMP-activated protein kinase (AMPK)-p38 pathways.	[74]
5	*Cornelian cherry (Cornus mas* L*.)*	Extracts of red and yellow from fruits	Modulate blood glucose levels and marker carbonyl oxidative stress	An *in vivo* study using rats induced by streptozotocin	•Extracts of red and yellow fruits of *Cornus mas* L. significantly lowered blood glucose by 7.1 and 8.6 mmol/L, respectively. •Similarly, in evaluating oral glucose tolerance tests, after the administration of extracts to diabetic rats, the blood glucose levels gradually reach fasting glucose. •On the other hand, glycated hemoglobin incredibly showed no changes in rats treated with extracts.	[70]
6	*Tiliacora triandra*	Ethanol extract	Insulin sensitizer and insulin secretagogue	An *in vivo* study using diabetic rats induced with high-fat diet (HFD)/streptozotocin (STZ)	•Blood glucose levels in rats treated with extracts at 100 and 400 mg/kg BW significantly declined compared to untreated rats. •The effect was correlated with the increased insulin levels in the treated rats, marked by lowering insulin resistance and improving beta cell function. •Furthermore, it was supported by the improvement of the morphology and architecture of Langerhans islets in groups treated by entities of extracts. •The other marker to evaluate the antidiabetic property of extract was Hb1Ac levels, significantly markedly lowering in treated groups.	[170]
7	*Citrus junos* Tanaka or Yuja	Ethanol extract from Yuja peel	Increases glucose uptake via AMPK and PPAR-γ signaling pathways	•An *in vitro* study in C2C12 myotubes •An *in vivo* in mice fed a high-fat diet	•The ethanol extract of Yuja peel (YPEE) contains flavonoids in which hesperidin is the major compound. •YPEE, in a dose-dependent manner, dramatically stimulated glucose uptake by stimulating the phosphorylation of AMPK and transcriptional activity of PPAR-γ.	[139]
			Decreases liver fat contents, triglyceride serum, and total cholesterol levels•		To clarify the antiobesity effect of YPEE, some parameters were measured. Interestingly, aside from regulating the AMPK and PPAR-γ signaling pathways, administration YPEE to high-fat diet groups dramatically decreased body weight, liver fat contents, triglyceride serum, and total cholesterol levels compared to the untreated group.	
8	*Syzygium cumini*	Aqueous extract from seed	α-amylase and α-glucosidase inhibitor	An *in vitro* α-amylase and α-glucosidase inhibitory assay	*Syzygium cumini* kernel phenolic (SCKP) extract offered potential antioxidant activity and antidiabetic as α-amylase and α-glucosidase inhibitor leading to the inhibition of glucose absorption in the intestine.	[168]
9	*Passiflora edulis*	Hydroethanolic extract 70% from leaves	•Modulates blood glucose level and HBA1c •Decreases blood cholesterol levels	An *in vivo* study using rats induced by alloxan	•Administration of extract in diabetic rats significantly decreased blood glucose and HBA1c. Flavonoid compounds presented in the extract alleviated the glycemic levels in rats. •*P. educulis* extract was able to reduce total cholesterol and non-HDL cholesterol in serum. Yet there was no effect on triglyceride and HDL-C.	[223]
10	*Glycyrrhiza foetida* and *Amorpha fruticosa*	Amorfrutins	Activate nuclear receptor PPARγ (peroxisome proliferator-activated receptor gamma)	•An *in vivo* study using high-fat diet-induced obesity (DIO) C57BL/6 mice. •An *in vitro* study using murine 3T3-L1 cells and human primary adipocytes	•Amorfrutins are potent and selective nuclear receptor PPARγ modulators. Unlike other synthetic PPARγ agonists, including the thiazolidinediones, amorfrutin 1 has a desirable effect in protecting the liver by lowering liver fat accumulation by approximately 50%. •The evidence proving the possible mechanism of amorfrutins in averting undesirable effects in the liver is amorfrutin 1 interacts directly with the liver-specific nuclear receptor PPARα leading to modulation of PPARβ/δ pathways and consequently contributing to alleviating liver steatosis. •The investigation of the effect of amorfrutin 1 on insulin resistance in C57BL/6 mice induced by a high-fat diet showed that the treatment group with amorfrutins 1 at 100 mg∕kg∕d for 23 days experienced an increase in insulin sensitivity along with a decrease in blood glucose during oral glucose tolerance and intraperitoneal insulin sensitivity tests. •On the other hand, amorfrutin 1 dramatically decreased plasma triglycerides, free fatty acids, equivalent to synthetic drug, rosiglitazone	[288]
11	*Carapa guianensis*	7-deacetoxy-7-oxogedunin (CG-1) isolated from seeds	Adipogenesis and lipolysis inhibitors	An *in vitro* study using adipogenesis assay, and lipolysis assay in 3T3-L1 cells	•The presence of 7-deacetoxy-7-oxogedunin (CG-1) decreased intracellular triglyceride level, differentiated adipocytes dose-dependently, and lowered lipid accumulation in adipocytes. •Further, to clarify the mechanism of CG-1 underlying suppression of adipogenesis, the mRNA levels of adipogenic, lipogenic, and lipolytic genes were measured by quantitative PCR. The result demonstrated that the mRNA levels of the three types of genes were suppressed by CG-1. In contrast, adipocyte lipolysis was unaffected.	[174]
12	*Camellia sinensis, Astrocaryum aculeatum*	8-C-ascorbyl-(−)-epigallocatechin	α-glucosidase and protein tyrosine phosphatase-1B (PTB-1B) inhibitor	An *in vitro* study	•8-C-ascorbyl-(−)-epigallocatechin (AE) promoted its antidiabetic properties by inhibiting α-glucosidase with IC50 of 142.8 μM. This yield was higher than acarbose (IC50 = 250.2 μM). •In addition, the mechanisms proposed of AE in increasing glucose uptake are increasing the phosphorylation of the p-Akt and inhibiting the production of protein tyrosine phosphatase-1B.	[314] [167]
13	*Hovenia dulcis* Thunberg	Flavonoids	Modulate AKT1 and GSK3β pathways	An *in-silico* study using STRING 10.0 and STITCH 5.0 and merged with Cytoscape 3.4.0	Flavonoids are the major constituents in *Hovenia dulcis* Thunberg, which notably decreased blood glucose by enhancing glucose uptake into cells. Flavonoid compounds of *H. dulcis* stimulate glycogen synthesis by activating AKT1 and inhibiting GSK3β. Aside from modulating glucose uptake, the consequence of suppression GSK3β is decreased proinflammatory cytokine synthesis leading to an anti-inflammatory effect.	[55]
14	*Leea macrophylla*	Ethanol extract from root	Increases insulin secretion, stimulates glucose uptake in the liver, and activates glycogenesis	An *in vivo* study using fructose-fed STZ-induced rats	•Three primary compounds in ethanol extract from *Leea macrophylla* root or Hatikana extract (HKEx), including oleanolic acid, 7α, 28-olean diol, and stigmasterol, have been identified. Treatment of HKEx by using three variety doses in diabetic rats for three weeks significantly lowered blood glucose levels, enhancing liver glycogen and serum insulin. •On the other hand, several biological biomarkers were determined, such as aspartate aminotransferase (AST), alanine aminotransferase (ALT), creatinine kinase (CK-MB), and lactate dehydrogenase (LDH). All of these parameters decreased drastically. •In addition, the investigation of the interaction between compounds and several protein targets was carried out, and the study revealed that SOD1 and CAT as antioxidative enzymatic increased.	[212]
15	*Fadogia ancylantha* (Makoni tea)	Bidesmosidic oleanolic acid saponins	α-amylase, α-glucosidase, and lipase inhibitor	An *in vitro* assay α-amylase, α-glucosidase, and lipase inhibitory assay	•Bidesmosidic oleanolic acid saponins 1–3 were isolated from *Fadogia ancylantha* (Makoni tea) and these compounds have different activities in inhibiting α-amylase, α-glucosidase, and lipase. Compounds 1–3 strongly inhibited α-glucosidase with IC50 values of 160, 170, and 190 μM, respectively. •Compounds 2–3 inhibited lipase with IC50 values of 190 and 200 μM, respectively; however, there was no activity in the inhibition of α-amylase. •On the other hand, only compound 1 inhibited α-amylase with an IC50 value of 180 μM. Interestingly, the inhibition activity of three compounds was more vigorous than acarbose as a standard drug.	[75]
16	*Angelica decursiva*	Coumarin-derivatives	α-glucosidase and protein tyrosine phosphatase-1B (PTB-1B) inhibitor	•An i*n vitro* study α-glucosidase and PTB-1B assay •An *in silico* study α-glucosidase (PDB ID: 3A4A) with AutoDock 4.2	•Coumarins derivates, consisting of (+)-trans-decursidinol, Pd–C–I, Pd-C-II, and Pd-C-III, have been identified from *Angelica decursiva* that strongly binding to active site enzyme α-glucosidase and protein PTB-1B. •(+)-trans-decursidinol, Pd–C–I, and Pd-C-II presented competitive inhibition, while Pd-C-III used mixed-type inhibition to PTB-1B. •(+)-trans-decursidinol showed competitive type; Pd–C–I and Pd-C-II mixed-type; Pd-C-III displayed non-competitive type inhibition of α-glucosidase. •Vigorous inhibition activity against PTP-1B of (+)-trans-decursidinol, Pd–C–I, Pd-C-II, Pd-C-III with IC50 values of 2.33, 4.32, 6.17, 11.98 μM, respectively. •Moreover, IC50 values in inhibition of α-glucosidase were 11.32, 17.40, 24.74, and 36.77 μM, respectively.	[7]
17	*Euonymus alatus* (Thunb.)			An *in vitro* study α-glucosidase and PTB-1B inhibitory assay	Compounds 15, 20, and 23 were potent inhibitors on α-glucosidase with IC50 values of 10.5 ± 0.8, 9.5 ± 0.6, and 9.1 ± 0.5 μM, respectively. Moreover, compounds 6, 7, and 23 were non-competitive inhibitors and vigorously inhibited PTB-1B with IC50 values of 13.7 ± 2.1, 5.6 ± 0.9, 13.7 ± 0.2 μM, respectively.	[120]
18	*Viburnum macrocephalum f.* keteleeri	Lignans glycosides			•Compound 4 exhibited potent action in inhibition α-glucosidase and PTB-1B with IC50 values of 9.9 ± 0.6 and 8.9 ± 0.5 μM, respectively. •Compound 4 displayed non-competitive inhibitors on α-glucosidase and mix-type inhibition against PTP-1B.	[317]
19	*Limonium gmelinii* (Willd.) Kuntze				Nineteen compounds were isolated from ethyl acetate extract of the roots of *Limonium gmelinii* (Plumbaginaceae), and compounds 1, 2, 14, and 18 strongly inhibited α-glucosidase with approximately range IC50 less than five μM. The activity of compounds 1–19 remarkably inhibited PTB-1B in the range IC50 of 1.71–50 μM.	[272]
20	*Hizikia fusiformis* (Harvey) Okamura				•Twenty-three compounds were isolated from the methanol extract of *H. fusiformis*. Incredible activity as inhibitor PTP-1B in compounds 1, 7, and 13 with IC50 of 6.59 ± 0.09, 4.86 ± 1.36, and 4.92 ± 0.01 μM, respectively. •Moreover, the inhibitor activity against α-glucosidase was more potent 3-fold than acarbose with IC_50_ of 48.05 ± 3.37, 34.85 ± 2.39, 43.90 ± 0.77 μM, respectively.	[233]
21	*Artemisia capillaris*	Esculetin, Quercetin, 3,5-Dicaffeoylquinic acid methyl ester•			Vigorous inhibitory activity of esculetin, quercetin, 3,5-Dicaffeoylquinic acid methyl ester against α-glucosidase was observed with IC50 values of 82.92, 58.93, and 86.95 μM, respectively; and protein tyrosine phosphatase-1B (PTB-1B) of 11.32, 17.40, 24.74, and 36.77 μM, respectively.	[183]
22	–	Hesperidin, naringin	α-glucosidase inhibitor	An *in vitro* study using p-nitrophenyl- D-glycopyranoside (p-NPG) as the substrate	Hesperidin and naringin possessed antidiabetic activity with remarkable inhibition against α-glucosidase with IC50 of 14.72 and 12.64 nM, respectively.	[261]
			Increase insulin secretion, decrease blood glucose and HbA1c	An *in vivo* study in HFD/STZ-induced diabetic rats	•Hesperidin and naringin at 50 mg/kg BW significantly decreased blood glucose and HbA1c levels and increased insulin levels in diabetic rats. •Blood glucose levels in diabetic rats treated with hesperidin and naringin were two-fold lower than the untreated diabetic rats, with levels of 124.03 ± 3.90 and 136.73 ± 3.19 mg/dl, respectively. •HbA1c levels were 5.85 ± 0.18 and 6.26 ± 0.17%, respectively. •Insulin levels were 21.55 ± 1.13 and 20.67 ± 1.08 μU/ml, respectively.	[169]
23	*Acacia auriculiformis*	Extract acetone from bark and empty pod	α-amylase, α-glucosidase inhibitors	An *in vitro* study using α-amylase and α-glucosidase assay	•Both extracts showed significant suppression of α-amylase and α-glucosidase with higher score inhibition. •Bark extract with score inhibition 64.55 ± 5.12% and 95.12 ± 4.75% on α-amylase and α-glucosidase at a concentration of 50 μg and 2.5 μg respectively. •Pod extract with score inhibition of 50.57 ± 5.12% and 79.1 ± 6.5% at a concentration of 50 μg and 5 μg on α-amylase and α-glucosidase, respectively.	[230]
25	–	Phenolic compounds			•Caffeic acid phenethyl ester and curcumin significantly inhibited α-glucosidase with IC50 values of 29.01 and 29.31 nM, respectively. • On the other hand, curcumin, rosmarinic acid, and isoliquiritigenin effectively inhibited α-amylase with IC50 values of 168.73, 137.36, and 169.52 nM, respectively.	[262]
26	*Chelidonium majus*	Chelerythrine	Activates PPAR-γ receptor	•An *in vivo* study using high-fat diet mice •An *in vivo* study in 293T cells	Chelerythrine significantly inhibited the CDK5-mediated phosphorylation of PPARγ and exhibited a unique mechanism in modulating glucose uptake and lipid metabolism.	[319]
27	–	Natural Prenylchalconaringenins and Prenylnaringenins	α-amylase, α-glucosidase inhibitors	•An *in vivo* study using diabetic mice induced with high-fat diet (HFD)/streptozotocin (STZ). •An *in vitro* study using α-amylase and α-glucosidase assay	•Geranylchalconaringenin exhibited more vigorous α-glucosidase inhibitory activity with IC50 of 1.08 μM, 50-fold higher than that of acarbose with IC50 of 51.30 μM. However, it presented moderate inhibitory activity against α-amylase with IC50 of 20.46 μM. •Geranylchalconaringenin at doses of 50 and 100 mg/kg BW deterred the increase of postprandial blood glucose levels.	[253]
28	*Tetracera indica* Merr.	Wogonin, norwogonin, and techtochrysin	Increase glucose uptake	An *in vitro* study in the 3T3-L1 cell	•Wogonin, norwogonin, and techtochrysin significantly induced adipogenesis with a similar effect to insulin and increased adipogenesis with similar action to rosiglitazone. •Wogonin and norwogonin greatly enhanced glucose uptake.	[93]
29	*Oroxylum indium*	Flavonoid glycosides, oroxins C and D	α-amylase, α-glucosidase, lipase inhibitors	*In vitro* study on α-amylase, α-glucosidase, lipase	Oroxins C and D inhibited lipase with IC50 of 190.1 ± 18.2 80.0 ± 9.5 μM, respectively. However, oroxins C significantly inhibited α-amylase two-fold higher than acarbose with IC50 of 210.3 ± 19.1 μM. Similarly, oroxins D with IC50 of 180.4 ± 25.7 μM was more potent in inhibition of α-glucosidase than acarbose.	[155]
30	*Bauhinia forficata* Link.	Kaempferitrin	Increase glucose uptake in soleus muscle	An *in vivo* study in alloxan-induced diabetic rats	•Glycogen content in soleus muscle diabetic rats after 3 h of treatment kaempferitrin dose 100 mg/kg BW drastically increased by 228% compared with the untreated diabetic rats. •The possible mechanism of kaempferitrin in regulating glucose uptake into cells is activating PI3K and MAPK pathways leading to the stimulation of insulin sensitization which upregulates GLUT4.	[38]
31	*Dillenia indica*	Kaempferol	Apoptosis cascade inhibition and increases insulin secretion	An *in vitro* study caspase-3 activity, intracellular ATP and cAMP, insulin secretion assay using isolated beta cells and human islets	•Isolated human islets with chronic high glucose were exposed to kaempferol for four days. The findings demonstrated that kaempferol inhibited cellular apoptosis by restoring anti-apoptotic protein AKT and Bcl-2, which was declined by chronic high glucose. •In addition, the caspase-3 activity was reduced in beta cells and human islets. •Furthermore, kaempferol ameliorated the suppression of cAMP and ATP production, leading to enhancing insulin synthesis.	[316] [167]
32	*Hypolepis punctata* (Thunb.) Mett.	Pterosin A	Increase glucose uptake via insulin sensitizer	An *in vivo* study using high-fat diet (HFD)–induced diabetic mice, and a dexamethasone-induced insulin-resistance (IR) mouse model	•Administration pterosin A at dose of 100 mg/kg BW orally for four weeks deterred hyperglycemia and glucose intolerance in diabetic mouse models. Moreover, treatment pterosin A at the dose of 100 mg/kg BW for one week restored the insulin intolerance in a dexamethasone-induced IR mouse model. The other parameters, such as HbA1c and serum insulin, were modulated near normal levels. •Glucose uptake was restored by upregulating GLUT4 translocation to transmembrane via MAPK signaling pathways.	[106]
33	*Eugenia punicifolia*	Aqueous extract from *Eugenia punicifolia* leaves (EEP)	•α-amylase, α-glucosidase, xanthine oxidase inhibitors •Free radical scavengers	An *in vitro* study in 3T3-L1 cells	•EEP showed significant inhibition against α-amylase, α-glucosidase, and xanthine oxidase activities with IC50 at 122.8 ± 6.3, 2.9 ± 0.1, 23.5 ± 2.6 μg/mL, respectively. •In addition, the EEP exhibited free radical scavenger activities by inhibiting free radicals of ABTS, DPPH, and O2 with IC50 at 10.5 ± 1.2, 28.84 ± 0.54, and 38.12 ± 2.6 μg/mL, respectively.	[162]
34	Grape	Grape-seed proanthocyanidin extract (GSPE)	Reducing body weight gain, adiposity, and liver steatosis	An *in vivo* study using cafeteria diet (CAF) high-fat/high-sucrose-induced syndrome metabolic in rats	•GSPE significantly lowered the food intake in CAF PRE (rats receiving preventive treatment of GSPE during ten days before cafeteria diet intervention) and CAF MONTHLY (rats receiving GSPE treatment during five days once per month simultaneously fed with cafeteria diet). •CAF PRE and CAF MONTHLY experienced a lowering of mesenteric adipose tissue weight at 21.0 ± 1.5 and 18.8 ± 1.4 g, respectively. Moreover, CAF MONTHLY rats showed a significant reduction in visceral adiposity compared to CAF rats at 14.0 ± 0.5%. •In CAF MONTHLY, GSPE proposed its effect by lowering the fat accumulation in the liver.	[243]
35	*Adansonia digitata* L.	Hydromethanolic extracts from fruit pulp and leaf	α-amylase, α-glucosidase, pancreatic lipase, and angiotensin-converting enzyme inhibitors	An *in vitro* enzymatic assay and study in SW-872 human liposarcoma cells	•Hydromethanolic extracts from *Adansonia digitata* L. fruit pulp and leaf showed remarkable inhibition activity against α-amylase, α-glucosidase, pancreatic lipase, and angiotensin-converting enzyme. •Leaf extract was the most potent in inhibiting α-amylase with IC50 of 0.10 mg/mL whereas fruit pulp extract inhibited α-amylase with IC50 of 97 mg/mL. •Leaf and fruit pulp extracts inhibited α-glucosidase with IC50 of 0.03 and 0.64 mg/mL, respectively, in a dose-dependent manner. •Leaf extract revealed the most potent inhibition of ACE and pancreatic lipase with IC50 of 0.08 and 1.85 mg/mL, respectively.	[47]
36	*Garcinia dulcis*	G. *dulcis* rind powder (CGD)	•Improve glucose tolerance and insulin sensitivity •Liver and cardioprotection	An *in vivo* study using high fat/carbohydrate diet (HFD) induced metabolic syndrome in rats	•The main compounds in CGD are garcinol, morelloflavone and citric acid. •Supplementation of CGD in high-fat/carbohydrate diet rats for eight and sixteen weeks showed the improvement glucose tolerance and insulin sensitivity. •CGD was able to repair the liver structure and function by lowering collagen deposition and reducing reduced aspartate transaminase activity in HFD rats. •The cardioprotective effect of CGD in HFD rats was exhibited by lowering systolic blood pressure, Left ventricular diastolic stiffness (κ), left and right ventricle wet weight, and recovery of cardiovascular structure and function.	[124]
37	*Phaseolus vulgaris* L.	Dry extract	•α-amylase inhibitor •Antihyperlipidemic •Antioxidant	An *in vivo* study using high-fat diet (HFD) induced metabolic syndrome in C57BL/6 mice	•*P. vulgaris* dry extract contains an alpha-amylase inhibitor and phytohaemagglutinin. •Treatment *P. vulgaris* dry extract 500 mg/kg BW for nine weeks in HFD mice showed significantly decreasing body weight and food intake compared with untreated groups. •Blood glucose, TG, total Cholesterol, and LDL in treated groups were significantly lower than in the untreated group in levels of 112.0 ± 4.4, 107.5 ± 9.3, 100.5 ± 7.6, and 38.4 ± 6.3 mg/dL, respectively. •*P. vulgaris* dry extract improved glucose tolerance and insulin resistance. •On histological examination, treated groups exhibited significant liver, cardiac, vascular, and adipose damage recovery. •Catalase and glutathione expression increased significantly, and NADH dehydrogenase and carbonylated protein decreased in the treatment groups with the extract.	[176]
38	*Cuscuta pedicellata*	Naringenin, kaempferol, aromadenderin, quercetin, aromadenderin-7-O-b-d- glucoside, taxifolin 7-O-b-d-glucoside	•Restore insulin resistance and glucose tolerance •Antioxidant activity	An *in vivo* study using a high-fat diet (HFD) induced obesity in rats	•Fasting blood glucose and plasma insulin levels in HFD groups treated with compounds and crude extract were significantly decreased compared with untreated HFD groups. •A lower HOMA-IR index indicated that treated groups with extract and compounds experienced restoration in insulin resistance. •SOD and catalase increased significantly, whereas thiobarbituric acid reactive substances (TBARS) decreased, indicating that the compounds and extracts opposed antioxidant mechanisms.	[175]
39	Mushrooms: *Lentinus edodes* and *Schizophyllum commune*	Ethanol and hexane extract s	α-amylase, α-glucosidase, and pancreatic lipase inhibitors	An *in vitro* study using enzyme assays	•Ethanol and hexane extracts from *Lentinus edodes* exhibited the most potent inhibition against α-glucosidase and pancreatic lipase. IC50 of ethanol extract in inhibiting α-glucosidase was 20.4 mg/mL, and hexane extract was 12.9 mg/ml, while IC50 of ethanol extract in inhibiting pancreatic lipase was 8.85 mg/mL, and hexane extract was 23.1 mg/mL. •The hexane extract from *Schizophyllum commune* exhibited a more significant inhibitory effect on α-amylase with IC50 15.3 mg/mL.	[300]
40	*Vernonia mesplilfolia* Less.	Ethanol and aqueous extracts			The ethanol extract was the most potent in inhibiting α-amylase and pancreatic lipase, with IC50 of 331.16 and 781.72 μg/mL, respectively. On the other hand, the aqueous extract exhibited the most potent α-glucosidase inhibitor with IC50 of 450.88 μg/mL.	[274]

**Table 2:** Prospective biological targets for natural products to manage heart-related diseases.

No	Source(s)	Compound or extract(s)	Mechanism of action(s)	Experimental model(s)	Key findings	Refs
1	*Rhizoma coptidis*	Berberine (BBR)	Induces the mitophagy-mediated HIF-1a/BNIP3 pathway	•An *In vivo* using rats subjected to MI/R surgery •An *In vitro* study in rat embryonic myocardium-derived cells H9C2	•BBR exhibited protective effects by inducing cardiomyocyte proliferation, inhibiting cardiomyocyte apoptosis, and activating the mitophagy-mediated HIF-1a/BNIP3 pathway. •CK-MB, LDH, and AST levels in all treated I/R groups with BBR at 300 mg/kg once a day for three consecutive days significantly decreased compared with untreated I/R groups.	[323]
			•Activates the AK2/STAT3 signaling pathway •Diminishs ER stress-induced apoptosis		•Administration of BBR several days before the cardiac injury was able to alleviate MI/R injury by activation of JAK2/STAT3 signaling. •Activation of JAK2/STAT3 prevented mitochondrial oxidative damage induced by myocardial ischemia. •BBR at 50 μmol/L significantly reduced SIR-induced cell apoptosis, oxidative stress, and ER stress in H9C2 cells.	[318]
			Modulating AMPK activity in both non-ischemic areas and risk areas of the heart	•An *In vivo* using rats subjected to MI/R surgery •An *In vitro* Isolated heart perfusion	•IR group treated with BBR exhibited rate of death (%), premature betas (times), last time of VF(s), and last time of VT(s) were significantly decreased compared with the untreated IR group with values of 10, 108.5 ± 14.1, 5.1 ± 1.7, and 4.1 ± 1.1, respectively. •In addition, infark size (IS) at risk area and Left Ventricular area in the group treated with BBR 100 mg/kg were significantly different from the untreated IR group. •BBR significantly decreased AMPK protein concentration and the ratio of ADP/ATP and AMP/ATP in AAR. Conversely, BBR significantly increased AMPK protein concentration and the ratio of ADP/ATP and AMP/ATP in NIA compared with controls.	[41]
				An *in vivo* study using ischemia-reperfusion injury in a rat model of type 2 diabetes	•DMIR, diabetic ischemia-reperfusion group, treated with BBR exhibited rate of death (%), premature betas (times), last time of VF(s), and last time of VT(s) were dramatically different from untreated DMIR. •BBR treatment enhanced AMPK activity and the ratio of ADP/ATP and AMP/ATP in non-ischemic areas. •Pretreatment with BBR stimulated protein kinase B (AKT) phosphorylation and suppressed glycogen synthase kinase 3b (GSK3b) activity in non-ischemic areas.	[40]
2	*Aralia elata*	Total saponins of Aralia elata (Miq) Seem (AS)	Modulate contractile function and intracellular calcium via activation PKCε phosphorylation	•An *in vitro* study using isolated rat ventricular myocytes •An *in vivo* study in dog models	AS showed positive effects in treating myocardial ischemia/reperfusion injury by exerting its mechanism to improve coronary blood flow, decrease oxygen consumption and heart workload with several actions, maintain the contraction and relaxation of myocytes, and activate PKCε, a Ca2þ-independent PKC isoform.	[284]
			Inhibit endoplasmic reticulum stress-related apoptosis	An *in vivo* study in myocardial I/R injury rats	•AS significantly reversed the pathological progress of myocardium, minimized infarct size, recovered the activities of Ca2+-Mg2+ -ATPase, Na + -K + -ATPase, sarcoplasmic reticulum Ca2+-ATPases (SERCA), and calcineurin (CaN). •Bcl-2 as anti-apoptotic was increased to prevent Bax oligomerization. •The expression of GRP78, C/EBP homologous protein (CHOP) was decreased, the subsequent biomarker oxidative stress (MDA) was reduced, and SOD was increased.	[285]
			Activate PI3K/Akt pathway and inhibition of MAPKs family	An *in vivo* study using lipopolysaccharide-induced cardiac dysfunction mice	•In immunohistochemistry, the infiltrated leukocytes significantly decreased in myocytes mice treated with AS 140 mg/kg BW compared with untreated mice. It correlated with reducing TNF-α, interleukin (IL)-1b, and IL-6 levels and preventing NF-kB activation. •LDH, CK, AST, and cTnI were decreased in treated mice. •ROS level was reduced as a result of downregulating LPS-mediated NOX2 expression. •The other mechanism of AS in treat LPS-induced cardiac dysfunction is significantly activated PI3K/Akt signaling pathway and inhibition of MAPKs family.	[43]
		Elatoside C	•Activates the STAT3 pathways •Reduces ER stress-associated apoptosis	An *in vitro* study in hypoxia/reoxygenation (H/R)- induced H9c2 cardiomyocyte injury	•Elatoside C significantly protected H/R-induced cell death by maintaining cell viability, stabilizing mitochondrial membrane potential, reducing mitochondrial ROS, and suppressing apoptotic cardiomyocytes. •On the other hand, apoptosis markers such as GRP78, CHOP, Caspase-12, and JNK were greatly suppressed. These data were supported by increasing STAT3 phosphorylation and an increase of Bcl2/Bax ratio.	[283]
3	*Brassica oleracea* var. *capitata rubra*	Anthocyanin	•Antioxidant •Cardioprotective	An *in vivo* study using atherogenic (ATH) diet-induced hypercholesterolemia and related cardiac in rats	•CK, CK-MB, and LDH in groups treated with anthocyanin-rich red cabbage extract at a dose of 100 mg/kg BW a day for eight weeks were significantly different from the untreated ATH group with levels of 54.17 ± 8.09, 82.50 ± 9.28, 98.10 ± 9.31 U/L, respectively. •In addition, MDA level was 9.00 ± 0.66, and SOD and CAT levels were 30.89 ± 1.45, 7.88 ± 0.19 U min−1 mg−1 protein, respectively. All of these parameters were significantly different from untreated ATH groups.	[226]
4	Songling Xuemaikang Capsule (SXC) (*Puerariae thomsoni, Pinus massonana,* and *powdered nacre*)	Songling Xuemaikang Capsule (SXC)	Inhibits of cardiac hypertrophy via CaMKIIδ and ERK1/2 pathways	•An *in vivo* study using an iso-induced cardiac remodeling model in rats •An *in vitro* study using H9C2 rat cardiomyocytes	•SXC suppressed the expression of CaMKIIδ, and the phosphorylation of ERK1/2, leading to inhibiting expression of GATA4 protein in the nucleus and brain natriuretic peptide in serum. •Moreover, left ventricular diastolic posterior wall thickness in the SXC group was significantly decreased. •The cardiac hypertrophy indicator HW/BW was decreased at dose-dependently.	[209]
5	*Beta vulgaris*	Betanin	Sentrin-specific protease −2 (SENP2) inhibitor	An *in-silico* study (PDB ID: 1TH0)	Betanin showed low toxicity, high binding energy, and hydrogen bonds to the SENP2 active site with low RMSD.	[255]
6	Wuwei Yuganzi San (WYS)	Sennoside D, quercetin, and procyanidin B-5,3’-O-gallate	Inhibiting of several crucial protein targets of CHD such as, ADAM17, AKR1C2, ALB, AKT1, and ADH1C	An *in-silico* study using AutoDock Vina software	The compounds showed binding affinity to protein targets, approximately < -10 kcal/mol, offered the promising therapeutic CHD.	[311]
7	*Allium sativum, Peganum harmala,* and *Berberis vulgaris*	Ethanol extract from *A.sativum* and *P. harmala,* and Methanol extract from *B. vulgaris*	Restoration of left ventricular remodeling, decreasing hs-CRP and NT-ProBNP	An *in vivo* study using isoproterenol-induced heart failure in rats	•Treatment ISO rats using *A. sativum, P. harmala,* and *B. vulgaris* exhibited heart weight/Body weight significantly different from ISO untreated rats. •Furthermore, extracts exhibited therapeutic effects by decreasing left ventricular end-diastolic/systolic diameters (LVED/Sd), NT-ProBNP and hs-CRP values, and increasing the ejection fractions.	[134]
8	*Terminalia arjuna* (Roxb.)	Lyophilized aqueous extract of stem bark	The extract modulated ERK/Akt, ER stress marker Grp78, and epigenetic regulator HDAC5.	An *in vivo* study using isoproterenol-induced cardiac hypertrophy in rats	•Down- and up-regulation of several proteins by isoproterenol was remarkably restored by the extract. •The other markers of cardiac hypertrophy, such as, heart-to-body weight ratio, interventricular septal and left ventricular posterior wall diameters, β-MHC, Sk.α Actin-1, BNP, and TGF-β2 were greatly restored. •The extract modulated ERK/Akt, ER stress marker Grp78, and epigenetic regulator HDAC5 and reversed to baseline.	[147]
9	*Radix salviae* Milthiorrhizae	Salvianic acid A (SAA) as a water-soluble fraction	Inhibite L-type calcium channels and decreasing myocardial contractility	An *in vivo* study using iso-induced myocardial ischemia injury in rats	Low and high doses of SAA inhibited cell shortening by 33.48 ± 0.75%, significantly reduced CK and LDH levels, inhibited L-type calcium channels in a dose-dependent manner, and histopathology of rat hearts were in normal structures.	[248]
10	*Cissampelos pareira*	Ethanol extract from root	Antioxidant activity and ameliorating calcineurin activity	An *in vivo* study using isoproterenol-induced cardiac dysfunction in rats	•Co-treatment CIS 200 mg/kg BW daily for 30 days proposed cardioprotective effects by regulating several cardiac dysfunction markers. LDH and TBARS levels significantly decreased at 437.65 ± 22.12 U/L and 7.52 ± 0.27 μM/L, respectively. In contrast, The GSH was increased at a 3.11 ± 0.11 μM/L level. In addition, NO, HW/BW, and calcineurin activity were lowered. •Antioxidant enzymes, such as catalase (CAT), superoxide dismutase (SOD), glutathione peroxidase (GPx), glutathione reductase (GR), and glutathione-S-transferase (GST) levels were significantly enhanced compared to untreated rats.	[244]
11	*Salvia miltiorrhiza*	*Salvia miltiorrhiza* hydrophilic extract (SMHE)	Antioxidant activity	A clinical study in diabetic patients with chronic heart disease (CHD)	•After treatment with SMHE 5 g twice daily for 60 days, GSH levels, SOD, and GSSG-R activities in patients were significantly higher than in the placebo group. •In contrast, the malondialdehyde (MDA) level in the treatment group was significantly lower than in the placebo group on the 30th day.	[211]
12	*Phyllanthus tenellus*	pino- cembrin-7-O-[3′′-O-galloyl-4′′,6′′-(S)-hexahydroxydiphenoyl]- α-D-glucose (P7OG)	Inhibit platelet aggregation, vasorelaxation, protection vascular disorders	An *in vitro* study using G-6-P, vascular reactivity, aggregation platelet assays.	P7OG greatly inhibited glucose-6-phosphatase, ADP, collagen with IC50 at 17.20, 26, 61 μM, respectively. In addition, P7OG showed remarkably inhibition effect on the G-6-Pase (83%) assayed in intact microsomes.	[73]
13	*Abies alba*	Silver fir trunk extract (SFTE)	Antiarrhythmia, vasoralaxan, antioxidant	An *in vivo* study using ischemic-reperfused isolated heart rats	SFTE significantly decreased lactate dehydrogenase (LDH) release rate, increased coronary flow rate, and restored arrhythmias duration by 80%, compared to untreated group during the reperfusion period.	[65]

**Table 3:** Prospective biological targets for natural products to manage cancer.

No	Source(s)	Compound or extract(s)	Mechanism of action(s)	Experimental model(s)	Key findings	Refs
1	*Arthrospira platensis*	Aqueous extract	•Antiproliferative •Modulate apoptosis in cancer cell	•An *in vitro* study using the human Caucasian non-small-cell lung adenocarcinoma A549 cell line and human foreskin fibroblast (HFF). •An *in vitro* study using MTT assay.	•Extract treatment to cancer cell lung A549 and HFF demonstrated that MDA and LDH levels in A549 cells increased significantly leading to an increase in the apoptotic process. •The cell cycle decreased significantly in the G1 phase of A549 cells, indicating that the cell cycle stopped in the G1, and it prevented entering phase M. As a result, the proliferative process was decreased in the A549 cell line. •In contrast, treatment with extracts showed no change in the necrosis process in both cells.	[256]
2	*Calotropis gigantea*	Dichloromethane extract (CGDCM)	Promote apoptosis through the mitochondria-dependent pathway	An *in vitro* study using human colorectal carcinoma HCT116 (CCL-247, ATCC, USA) and colorectal adenocarcinoma HT-29 (HTB-38, ATCC, USA).	•Cytotoxic effects of CGDCM on HCT116 and HT-29 cells were higher than 5-fluorouracil with IC50 of 5.9 ± 0.62 and 44.0 ± 4.06 μg/mL, respectively. •Combinations of CGDCM (4, 8, and 10 μg/mL) with 5-FU (5 μM or 0.65 μg/mL) significantly enhanced the induction of apoptosis compared with either of the drugs used alone. •The expression of the pro-apoptotic protein levels, such as c-caspase 3, was significantly increased in HCT116 cells treated with CGDCM and combination CGDCM with 5-FU. In contrast, the levels of anti-apoptotic (Bcl-2) and ATP were decreased. •CGDCM (4 and 8 μg/mL), 5-FU (5 μM or 0.65 μg/mL), and combinations simulated the increasing ROS levels. As a result, the apoptotic process was stimulated.	[293]
3	*Bombax buonopozense*	Ethanol extract	•Antioxidant •Antiproliferation	•An *in vitro* study using P815 murin lymphoblast-like mastocytoma cell line. •An *in vitro* study using MTT assay.	•The extract contained flavonoids, tannins, alkaloids, and triterpenes. •Ethanol extracts showed considerably potent scavenging effects on DPPH radicals with IC50 values of 10 μg/mL. •Ethanol extract exhibited moderate inhibition on P815 cells in a dose above 200 μg/mL with IC50 of 74 μg/mL compared with cisplatin IC50 with IC50 of 4 μg/mL).	[266]
4	*Glycosmis parva*	Arborinine	Inhibits the growth of tumor	An *in vitro* study using adriamycin-resistant SGC-7901 (SGC-7901/ADR) cell line, Vincristine- resistant SGC-7901 (SGC-7901/VCR) cell line, Paclitaxel-resistant MGC803 (MGC/PTX) cell line.	•Arborinine exhibited a powerful inhibitory effect in SGC-7901, SGC-7901/ADR, SGC-7901/VCR, and MGC803 (MGC/PTX) with IC50 of 1.96, 0.24, 1.09, and 1.32 μM, respectively. •Arborinine significantly decreased cell viability in gastric cancer cells and drug-resistant gastric cancer cells for 48 h in a dose-dependent manner.	[46]
5	*Moringa oleifera*	Soluble extract from leaves	•Induces of apoptosis •Antioxidant •Antiproliferative	An *in vitro* study using A549 lung adenocarcinoma cells	•The extract exhibited considerably inhibitory effects on the proliferation of A549 lung adenocarcinoma cells in a dose/time-dependent manner. •The extract exhibited potent induction of protein caspase-3 expression, stimulating apoptosis cascade. •The extract decreased the level of intracellular levels in a concentration-dependent manner.	[157]
6	Sponge *Hyrtios* sp.	Methanol extract	Induces apoptosis via activation p53 and inhibition JNK pathway	An *in vitro* study using human colorectal carcinoma RKO (CRL-2577) and RKO-E6 (CRL-2578) cells	•The extract was able to induce a mitotic catastrophe •The extract increased the expression of p21 protein, which correlated to increasing of p53 in RKO cells. •In addition, the presence of extract suppressed JNK protein expression in RKO and RKO-E6 cells	[126]
7	*Juniperus indica* Bertol	The crude extract of the liquid oil	Antiproliferative effect by interfering with Akt/mTOR signaling pathway	An *in vitro* study using OECM-1 human gingival squamous cancer cells line.	Induces apoptosis via activation p53 and inhibition JNK pathway	[107]
8	*Rhaponticum carthamoides* (Willd.)	Methanol extract from root	Induces mitochondrial dysfunction	An *in vitro* study using leukemia cells (K-562 and CCRF-CEM) and lung adenocarcinoma cells (A549).	•The extract significantly decreased viability cells in a dose-dependent manner. •Mitochondrial membrane potential was disrupted and extract significantly increased mitochondrial DNA lesions in ND1 and ND5 genes and DNA damage in the TP53 gene.	[245]
9	*Xanthium strumarium*	Chloroform and methanol extracts from fruit	Inhibit autophagy-related (ATG) proteins	An *in vitro* study using ATG4B cleavage assays.	•Extracts significantly suppressed the cell invasion, migration, and live cells in colorectal cancer cells. •The presence of extracts significantly inhibited cell migration. •The extracts decreased viability cells in a dose-dependent manner. •Extracts increased luciferase activity compared with cells without treatment, indicating that autophagy in cancer cells was suppressed. •The levels of MAP1LC3-II protein were increased, indicating that extracts inhibited autophagy proteolytic activity.	[39]
10	*Litchi chinensisSonnnerat*	n-butyl alcohol extract of Litchi seed (NLS)	•Induces cell apoptosis by inhibiting Akt/GSK-3β signaling pathway and activating the intrinsic apoptotic pathway •Inhibits cell migration	An *in vitro* study using prostate cancer cell lines PC3, DU145, RM1, and C4–2B	•NLS considerably inhibited the growth and proliferation of prostate cancer cells in a concentration-dependent manner. •NLS activated the intrinsic apoptotic pathway by inducing the cleaved caspase-9 in cells and cleaved Caspase-7. •NLS suppressed the expression of anti-apoptotic Bcl2 and increased pro-apoptotic protein Bax in both PC3 and DU145 cells. •NLS significantly inhibited the phosphorylation of Akt and GSK-3β in both PC3 and DU145 cell lines. •NLS promoted cell cycle arrest at the G1/S phase through suppression of cyclin-dependent kinases (Cdks) and upregulation of CDK inhibitor	[88]
11	*Annona muricata* L.	Ethanol extract from leaves	•Induces cell apoptosis •Decreases cell viability	An *in vitro* study using liver cancer HepG2 cells and colon cancer HCT116 cells	•The extract significantly decreased cell viability in both HepG2 and HCT116 cells in a concentration-dependent manner. •The extract remarkably upregulated the expression of HSP70, GRP94, DPI-related protein 5, Bip, CHOP, and phosphorylation of PERK and eIF2α in the cancer cell line.	[160]
12	*Neptunia oleracea Lour* (water mimosa)	Methanol extract	•Induces cell apoptosis •Antiproliferation	An *in vitro* study using jurkat (acute T cell leukemia) and MV-4-11(biphenotypic B myelomonocytic leukemia) cell line.	•The extract significantly induced apoptosis in cancer cells by suppressing Bcl-2, c-Myc, and pERK1/2 protein levels. In contrast, cleaved PARP was increased.	[27]
13	*Cyanthillium cinereum* (L.)	Sesquiterpene lactones	•Cytotoxicity activity •Inhibit DNA replication by inducing S-phase arrest •Induce cell apoptosis	An *in vitro* study in 786-O cell line, K-562 leukemic cell line, and MCF-7 breast cancer cell line	•Compound 1 at 12.5 and 25.0 μg/mL concentrations significantly induced S phase arrest with IC50 of 12.02 and 13.3%, respectively, compared to the control cell. •Compound 1 increased ROS production in 786-O cells in a time-dependent manner but gradually was weaker after incubation for 2 h. •Compound 1 significantly increased LDH release in a time/concentration-dependent manner.	[60]
14	*Tourneuxia variifolia*	Ethyl acetate (EtOAc) and n-butanol (n- BuOH) extracts	Inhibit the activity of HeLa cells	An *in vitro* study using human cervical adenocarcinoma (HeLa) cell line	•EtOAc and n-BuOH extracts contained high quantities of phenolic compounds. •The EtOAc extract showed potent anticancer activity with IC50 of 46.797 ± 0.060 mg/mL.	[309]
15	*Tapinanthus sp.* (Loranthaceae)	•Methanol extract from leaves •Flavonoid glycoside (compound 3)	Inhibit proliferation	An *in vitro* study using glioblastoma (U87MG, C6) and prostate (PC-3) cancer cells	•The methanol leaves extract exhibited great anticancer activity in U87 with IC50 of 21.40 mg/mL and PC-3 cells with IC50 of 10.26 mg/mL. •Compound 3, the most potent, inhibits the proliferation of C6 and PC-3 cells with IC50 of 38.84 and 21.33 mM, respectively.	[81]
16	*Xylocarpus granatum*	Ethyl acetate extract from leaves	•Antioxidant •Inhibits the activity of cancer cells	An *in vitro* study using HeLa, T47D, and HT-29 cell line	•Antioxidant activity was examined using DPPH assay, and the extract showed intermediate antioxidant activity with IC50 of 84.93 ± 12.93 ppm. •Cytotoxicity of extract in HeLa, T47D, and HT29 was determined using MTT assay and exhibited IC50 of 42.50 ± 36.56, 559.57 ± 857.79, 77.76 ± 66.70 ppm, respectively. •Fraction 5 of the extract revealed the most potent inhibition against HT-29 with IC50 of 23.12 ppm.	[53]
17	*Diospyros kaki* L.	Total flavonoids from persimmon leaves (FPL)	•Inhibit proliferation and migration of cell •Induce cell apoptosis by activation of oxidative stress and mitochondrial-related apoptotic.	An *in vitro* study in prostate cancer PC-3 cells	•FPL induced a cytotoxic effect in a concentration-dependent manner starting at 12.5–100 μg/ml. •FPL-induced cell apoptosis was marked by increased ROS, MDA, nitrite, iNOS activity, and mitochondrial membrane permeability. •FPL significantly suppressed protein Bcl-2, increased BAX and cleaved caspase-3, and released cytochrome *c*. •FPL significantly inhibited the migration of PC-3 cells.	[62]
18	*Tephroseris kirilowii* (Turcz.) Holub.	Isorhamnetin (IH), genkwanin (GN), acacetin (Aca)	Induce apoptosis by reducing PI3Kγ -p100 mediated PI3K/AKT/mTOR/p70S6K/ULK signaling pathway.	•An *in vitro* study using human breast cancer cells (MDA-MB-231) •*In-silico* using Surflex-Dock in SYBYL2.0	•IH, GN, and Aca inhibited cell proliferation in a concentration-dependent manner associated with cell cycle arrest at the G2/M phase. •IH, GN, and Aca induced cell apoptosis due to decreased Bcl-2 and Bcl-xL and increased levels of p53. •IH, GN, and Aca inhibit expression of PI3K/AKT/mTOR/p70S6K/ULK1, as well as PI3Kγ. •IH, GN, and Aca-induced autophagic correlated with decreasing of p62 and increasing in levels of ATG5. •The docking results demonstrated that IH, GN, and Aca are able to bind to the specific functional catalytic amino acids of PI3Kγ with hydrophobic interaction, such as LYS-833 and ASP-964.	[310]
19	*Artemisia aucheri* Boiss.	Methanol extract from leaves	•Cytotoxicity •Induces apoptosis •Inhibits migration cell	An *in vitro* study using HT29 colon cancer cells	•The cytotoxicity effect of the extract was dose-dependent. The higher concentration showed lower cell viability. •The level of malondialdehyde was significantly increased in the treated cells with the extract. •The extract significantly induced apoptosis and inhibited the migration of cells.	[6]
20	*Calligonum comosum* (L’Her)	Methanol fruit hairs extract (MFH)	•Antiproliferation •Induces apoptosis	An *in vitro* study using human hepatocarcinoma cells (HepG2)	•MFH exerted potent antiproliferation activity with IC50 of 10.4 mg/ml. •MFH induced overexpression of mRNA transcript levels of gen p53, caspase-3, and Bax as pro-apoptotic. In contrast, the level of Bcl-2, an anti-apoptotic marker gene, was suppressed.	[9]
21	*Bombax buonopozense*	Ethanol extract from stem bark	•Antiproliferation •Antioxidant	•An *in vitro* study in P815 murin lymphoblast-like mastocytoma cell line using the MTT assay •Antioxidant activity was measured by the 2,2’-diphenyl-1- picrylhydrazyl (DPPH) free radical assay	•The extract showed moderate inhibitory activity against P815 in a dose-dependent manner in which IC50 above 200 μg/mL was 74 μg/mL. •The antioxidant activity revealed IC50 of 10 μg/mL at a concentration of 220 μg/mL.	[266]
22	*Raphanus sativus* L.	Ethanol extract from seed	•Inhibits proliferation and cell migration mediated by the β-catenin signaling pathway •Induces apoptosis	An *in vitro* study using oral squamous cell carcinoma (KB and KB^CD133+^)	•The extract decreased β-catenin activity, expression, and nuclear translocation in a dose-dependent manner. •The extract could induce apoptosis by upregulating PARP, Bax, and downregulating Bcl-2. •In addition, the p-GSK-3b level and p-GSK-3b/t-GSK-3b ratio were significantly decreased dose-dependently, leading to induced apoptosis.	[3]
23	*Orobanche crenata*	Methanol extract	•Antioxidant •Cytotoxic •Induces apoptosis	An *in vitro* study using hepatocellular carcinoma (HepG2), human prostate cancer (PC3), human breast adenocarcinoma (MCF-7), and human colon carcinoma (HCT-116)	•The extract exhibited potent antioxidant activity. •The extract revealed a remarkable cytotoxic effect on HepG2, PC3, MCF-7, and HCT-116 cells with IC50 values of 30.3, 111, 89.6, and 28.6 mg/mL, respectively. •The presence of extract in a concentration-dependent manner increased LDH release, leading to membrane cell damage in HCT-116 cells. •Extract activated caspase-3 activity in HCT-116 cells to induce cell apoptosis.	[96]

**Table 4:** Natural products displaying potency as anti-diabetes mellitus, -obesity, -heart-related diseases, and -cancer by specifically modulating excessive effects of proinflammatory cytokines.

No	Source(s)	Compound or extract(s)	Mechanism of action(s)	Experimental model(s)	Key findings	Refs
Natural products to manage diabetes mellitus and obesity						
1	Psidium guajava	Total triterpenoids of leaves extract	Inhibitor of proinflammatory cytokines by NF-κB pathway	An *in vivo* study using rats induced by a high-fat diet and streptozotocin to provide diabetic peripheral neuropathy	Significantly decreased serum blood glucose levels in rats and suppressed the expression of proinflammatory mediators via PI3K and Akt pathways.	(X [287].
Natural products to manage heart-related diseases						
2	Rhizoma coptidis	Berberine (BBR)	Suppressing NF-κB and JNK signaling pathways	•An *in vitro* study in H9c2 cells subjected to hypoxia/reoxygenation •An *in vivo* study using rats subjected to MI/R surgery	•Pretreatment of solid dispersion of BBR with sodium caprate (HGSD) at doses of 25 and 50 mg/kg significantly enhanced the recovery of cardiac LVDP 2-fold, cardiac output 2.5-fold, and decreased cardiac LVEDP 1.8-fold compared with the untreated group. •HGSD significantly suppressed the release of cTnI into the perfusate after ischemia-reperfusion. •The possible mechanism of HGSD pretreatment in inhibiting the production of TNF-α and IL-6 is related to diminishing JNK activation leading to decreasing translocation of NFκB into the nucleus.	[306]
3	Aralia elata	Total saponins of Aralia elata (Miq) Seem (AS)	Inhibit NF-kB activated by TNF-α and stimulating PI3K/Akt signaling pathway to regulate the pro- and anti-apoptotic	An *in vitro* study in endothelial Cell Injury induced by TNF-α using human umbilical vein endothelial cell (HUVEC)	•Inhibition of NF-kB activated by TNF-α was reported as a cytoprotective effect of AS. In addition, AS stimulated PI3K/Akt signaling pathway to regulate the pro- and anti-apoptotic protein Bcl-2/Bax and downregulate inflammatory factors such as IL-6, MCP-1, and VCAM-1.	[321]
4	Grape	Oligomerized grape seed proanthocyanidins (GSP)	•Inhibition of oxidative damage •Suppression of the ASK1/NF-kB signaling pathway	An *in vivo* study using an iso-induced cardiac remodeling model in rats	•The increase of HW/BW and LVW/BW as cardiac hypertrophy indicators was significantly diminished by the administration of GSP at all doses compared with untreated groups. •Treatment with GSP at doses of 100 and 150 mg/kg BW significantly inhibited NF-kB signaling pathway, decreased the expression of COX-2 protein, increased SOD, and suppressed MDA.	[324]
5	Salvia miltiorrhiza Bge. and Carthamus tinctorius L.	Danhong injection (DHI) contains 5-hydro- xymethylfurfural, Danshensu, protocatechuic acid, protocatechuic aldehydrate, caffeic acid, rosmarinic acid, lithospermic acid, salvianolic acid B, salvianolic acid A and salvianolic acid C	Anti-cardiac hypertrophic by modulating p38 and NF-κB pathway	•An *in vivo* study using iso-induced cardiac hypertrophy in rats •An *in vitro* study in H9C2 cells	DHI suppressed the elevation of P38 phosphorylation and activation NF-κB inhibiting translocation of p65 into the nucleus. The subsequent event is the restoration of cardiac hypertrophy induced by ISO.	[172]
6	–	Quercetin, luteolin and epigallocatechin gallate	•Antiinflammation •Inhibit apoptotic cascade • Regulate ROS production	An *in vitro* study using EA. hy-926 cells	•Quercetin, luteolin, and EGCG increased AMPK phosphorylation, decreasing TXNIP and NLRP3 inflammasome induction, leading to the downregulation of IL-1β, which strongly correlates with lowering caspase-3 activity •Regulation of eNOS and ET-1 expression in endothelial cells, inhibition IKKβ activation, leads to attenuated phosphorylation and downregulated gene expression of VCAM-1.	[296]
Natural products to manage cancer						
7	Gynura procumbens	Ethanol supernatant extracts (EEGS)	•Antiinflammation •Antiproliferation	An *in vivo* study using nanodiethylnitrosamine (nanoDEN)-induced mouse liver cancer	•The major constituents of G. procumbens are caffeoylquinic acid (CAC) and non-caffeoylquinic acid (n-CAC). •The incidence of tumors was significantly reduced in treatment with EEGS-L (10 mg/kg) and CAC compared with the untreated nanoDEN group. •Administration of EEGS-L, CAC, and n-CAC significantly lowered the inflammation scores. •Administration of EEGS, CAC, and n-CAC significantly suppressed COX-2, β-catenin, PCNA, HIF-1α expression induced by nanoDEN. •Administration of EEGS, CAC, and n-CAC significantly decreased mRNA levels of β-catenin, TNF-α, PPAR-γ, AP- 2, Smad-2, and TGF-β1 during tumorigenesis. •Administration of EEGS, CAC, and n-CAC remarkably reversed nanoDEN-induced submicroscopic structural changes in liver tissue.	[313]
8	*Citrullus lanatus* (Thunb.) Mansfeld	Lycopene	•Induces cell apoptosis •Induces elevation of proinflammatory protein	An *in vitro* study using adenocarcinoma cell line (A549 CCL-185™)	•Lyc W significantly induced the apoptotic cells and mitochondrial stress. •Increased intracellular ROS levels and translocation of NF-kB. •Increased elevation of IL-8 as a proinflammatory protein.	[61]

### 1.4 Natural products for the management of inflammation-related metabolic disorders

The process through which complex macromolecules like proteins, carbohydrates, and lipids are broken down into their constituent parts is called metabolism. When regular metabolic processes are hampered, it can lead to metabolic disorders. Diabetes mellitus, obesity, heart-related syndromes, and cancer are the metabolic disorders that are seen the most frequently [103]. Over the last decade, numerous efforts have been made to include natural products into drug development [18]. More than two-thirds of drug active ingredients are derived from natural sources [189].

#### 1.4.1 Natural products to manage diabetes

A lack of functioning β-cells in the Langerhans islets causes insulin resistance, which in turn causes high blood sugar levels to remain elevated and, eventually, diabetes mellitus [30,117]. "Diabetes Mellitus" is a phrase that was coined from the Greek language. In Greek, the word "Diabetes" means "a passer through," while the word "Mellitus" means "sweet." [239]. When the body stops producing or effectively utilizing insulin, it causes serious problems for the cardiovascular system, the blood vessel system, the eyes, and the kidneys. The prevalence of diabetes is rising rapidly, making it one of the world's leading health concerns. In 2019, the International Diabetes Federation (IDF) predicted that 463 million adults had diabetes; this number is expected to increase to 578 million by 2030 and to 700 million by 2045 [108,222]. In addition, roughly 374 million people worldwide had diabetes in 2017 but did not know it [108]. There are two distinct types of diabetes mellitus: type 1 and type 2. The immune system mistakenly attacks and destroys β-cells in response to environmental triggers such as chemicals [105] and viruses [115], resulting in T1DM. Therefore, exogenous insulin is essential for the management of type 1 diabetes [121]. About 10% of all diabetic patients suffer from this condition, which is particularly common in young people [21]. Unlike type 1, which typically manifests in childhood or adolescence, T2DM (also known as "non-insulin-dependent diabetes") develops in adulthood and is characterized by the body's inefficient use of insulin (known medically as "peripheral tissue resistance") [260]. Sunlight exposure in childhood was found to protect against the onset of T1DM [114]. A healthy lifestyle, including a nourishing food, exercise, increased physical activity, not smoking, and maintaining a moderate body weight, can help reduce the chance of developing T2DM [5, 17, 303]. Though there are medications capable of curing T2DM, including metformin [232], sulfonylurea [113], and insulin [143] are the currently available scientifically proven synthetic anti-diabetic medications. Also, α-glucosidase inhibitors [131], thiazolidinediones [275], glucagon-like peptide-1 receptor agonists [205], pramlintide [101], and dipeptidyl peptidase-4 inhibitors [56] are some of the newer medications with little evidence supporting their use. Therefore, there is a lack of drugs that are both effective and have few unwanted side effects, such as severe hypoglycaemia [112], and in some conditions, they lack safety [200], so it is important to investigate alternative medicines for the management of diabetes. In most cases, the availability, affordability, and safety of alternative medicines would far outweigh their disadvantages [200]. Consistent efforts are being made to investigate diabetes and discover new therapeutic strategies, such as the identification of natural products with anti-diabetic effects [187], due to the disease's high prevalence and the lack of satisfactory treatment options. People with diabetes have used a wide range of alternative treatments to control their condition. Pre-clinical and clinical trials have been conducted on a variety of natural products for the treatment of diabetes.

**Fig. 5:**
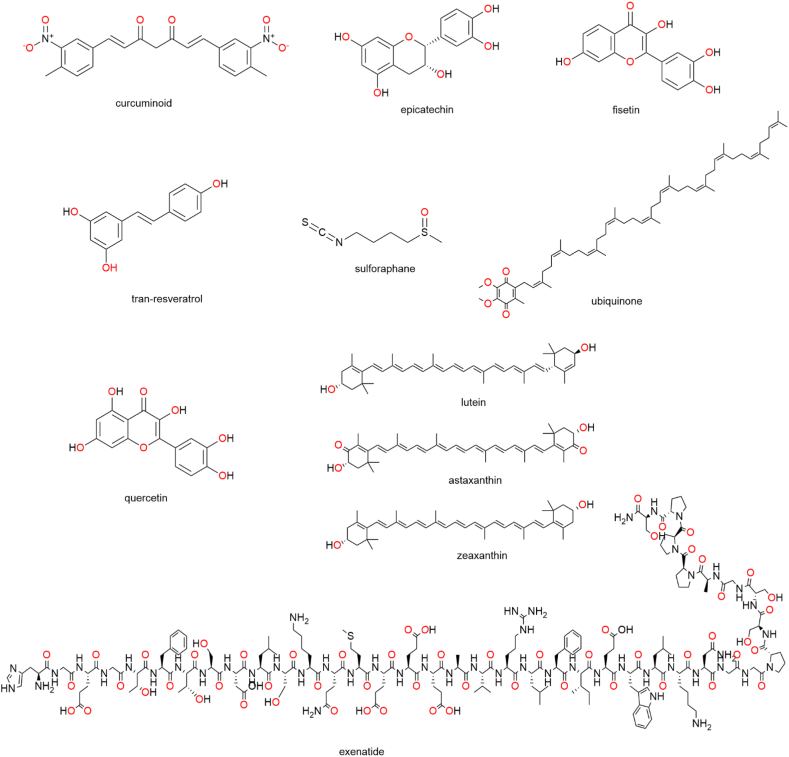
Natural compounds with antidiabetic potential and their chemical structures.

As shown in Table 5 and Fig. 5, many naturally occurring substances have the potential to aid in the control of blood sugar levels in diabetic patients. Mechanisms of anti-diabetic action include the suppression of digestive enzymes like α-glucosidase and α-amylase [216], changes in glucose uptake and the expression of glucose transporters [69], increased insulin secretion and pancreatic β-cell proliferation [150], suppression of insulin resistance [220], and regulation of oxidative stress [109]. Evidenced by the vast quantity of molecules with natural product origins that have undergone clinical trials, natural products remain a promising source for the development of novel therapeutics.

**Table 5:** Clinical trials of natural products with anti-diabetic activity (https://clinicaltrials.gov).

Compound	ClinicalTrials.gov Identifier	Type of study	Characteristics of patients (n)	Dose and time of treatment	Condition	Phase	Additional Refs
Curcuminoid	NCT02529982	randomized, double-blind, placebo-controlled trial	curcumin group (n = 25) meals or placebo group (n = 28)	1500 mg capsule for 10 weeks	type 2 diabetes	–	[100]
*Trans*-resveratrol	NCT01677611	randomized, placebo-controlled trial	n = 10	500 mg to a maximum of 3 g daily	type 2 diabetes	phase 1	
Resveratrol	NCT01354977	a placebo-controlled study	resveratrol group (n = 12) or placebo group (n = 8)	1,000 mg twice daily for 28 days	type 2 diabetes	phase 2	
Quercetin	NCT01839344	crossover, double-blinded, controlled trial	Quercetin, acarbose and placebo (n total = 19)	250 mg; oral single dose of 2000 mg	type 2 diabetes	phase 2	
Epicatechin	NCT02330276	double-blinded randomized	Each dose has n = 4	epicatechin 10 mg, 30 mg, or 100 mg	pre-diabetes	phase 1	
Sulforaphane	NCT02801448	randomized, double blind, placebo-controlled trial	sulforaphane group or placebo group; n = 103	sulforaphane-containing broccoli sprout extracts once daily for 12 weeks	type 2 diabetes	phase 2	
Ubiquinone	NCT02062034	randomized double-blind placebo-controlled study	ubiquinone group, antioxidant combination group, placebo; n = 40	400 mg daily of oral ubiquinone for 24 weeks	non-proliferative diabetic retinopathy, type 2 diabetes	phase 2	[97]
Lutein, astaxanthin, zeaxanthin, vitamin C, vitamin E, zinc copper	NCT03702374	randomized double-blind placebo-controlled study	antioxidant combination group and placebo; n = 132	antioxidant combination tablet once a day for 12 months	diabetic retinopathy	phase 3	[163,184,217]
Fisetin	NCT03325322	randomized double-blind placebo-controlled study	fisetin group and placebo; n = 30	20 mg/kg/day, orally for 2 consecutive days	diabetes mellitus, diabetic nephropathies, chronic kidney diseases	phase 2	
Exenatide	NCT02735031	randomized double-blind placebo-controlled study	exenatide group and placebo; n = 10	week 1–2: 5 μg twice daily; week 3–6: 10 μg twice daily (if tolerated)	type 1 diabetes, hypoglycemia	phase 2/3	
Exenatide	NCT01876849	open-label	N = 275	injection 5mcg or 10 mcg, twice daily	type 2 diabetes	phase 3

#### 1.4.2 Natural products as lipid-lowering agents

**Fig. 6:**
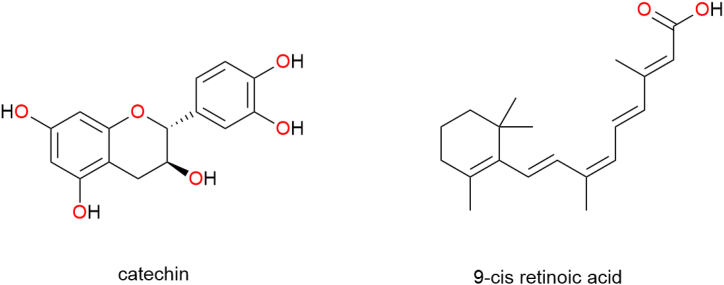
Natural compounds as lipid-lowering agent and their chemical structures.

Adipose tissue build-up to an unhealthy degree characterizes obesity [185]. It is one of the world's most serious public health issues, affecting people of all ages and genders and all races [118, 292]. Obesity is typically brought on by an inability to maintain a healthy balance between dietary intake and energy expenditure, which is controlled by a wide range of physiological mechanisms [51]. There was a significant increase from 1980 to 2013 in the global prevalence of overweight, with 36.9% of men and 38.0% of women being overweight that year [182]. 671 million people were found to be obese throughout the world in this survey [182]. BMI values between 25.0 and 29.9 kg/m^2^ and 30.0 kg/m^2^ are commonly used to define overweight and obesity, respectively [48,229]. Obesity is the result of a complex interplay between genetic predisposition, the built environment, and individual behavior [273,301]. Many diseases and conditions are linked to obesity, including metabolic syndrome [59], pulmonary diseases [227], dyslipidaemia [58], cancer [36,138], non-alcoholic fatty liver disease [294], hypertension [234], gastrointestinal diseases [79], and diabetes mellitus [10,141]. The rising rates of obesity-related illness and death also place a heavy financial burden on healthcare systems [153]. There are currently available synthetic anti-obesity drugs such as orlistat, a reversible inhibitor of lipase enzymes in the GI tract that can reduce fat absorption [102], and lorcaserin, a serotonin-2C receptor agonist that suppresses appetite and promotes satiety [33]. Therefore, numerous natural products have the clinical potential as lipid-lowering agents for obese and overweight people, as shown in Table 6 and Fig. 6.

**Table 6:** Clinical trials of natural products as lipid-lowering agents (https://clinicaltrials.gov).

Compound	ClinicalTrials.gov Identifier	Type of study	Characteristics of patients (n)	Dose and time of treatment	Condition	Phase	Additional Refs
Catechin	NCT00692731	randomized, double-blind, controlled study	catechin group and control group	500 mL/day of a beverage providing approximately 625 mg catechins	overweight, obesity	–	[100]
Polyphenols	NCT05255367	open label	n = 26	Daily consumption of 100 mL of commercial berry and pomegranate juice, 20 g dark chocolate, and 1 green tea for 2 months to see if diet supplementation with (poly)phenol rich foods worked.	overweight, obesity	–	
9-cis retinoic acid of *Dunaliella bardawil*	NCT00156169	randomized, double-blind, controlled study	Dunaliella group and control group n = 50	four Dunaliella capsules, providing 60 mg b-carotene per day after fibrate treatment	low HDL, cholesterol	phase 3	[23,235]
Exenatide	NCT01061775	open-label	n = 19	5mcg twice day for 4 weeks, then 10mcg twice daily for 20 weeks.	hypothalamic obesity	phase 1/2	[161]

#### 1.4.3 Natural products to treat heart-related diseases

Diseases of the heart and blood vessels are referred to as cardiovascular diseases (CVD) [78, 198]. The most frequent forms of cardiovascular disease are hypertension [140], coronary artery disease [218], cerebrovascular disease [206], angina pectoris [122], and atherosclerosis [77]. Risk factors for cardiovascular disease can be split into two groups: modifiable and non-modifiable risk factors [178]. Modifiable risk factors include insufficient physical exercise, an unhealthy diet, obesity, and a disordered lipid profile [228]; non-modifiable risk factors include smoking and high blood pressure [128]. There are some personal risk factors that cannot be changed, such as genes, sex, age, or family history [111]. Tobacco usage is associated with an increased risk of cardiovascular disease [145], lung disease [166], and cancer [95]. Numerous studies have demonstrated that CVD risk can be reduced with healthy eating, regular exercise, and smoking cessation [127, 252].

**Fig. 7:**
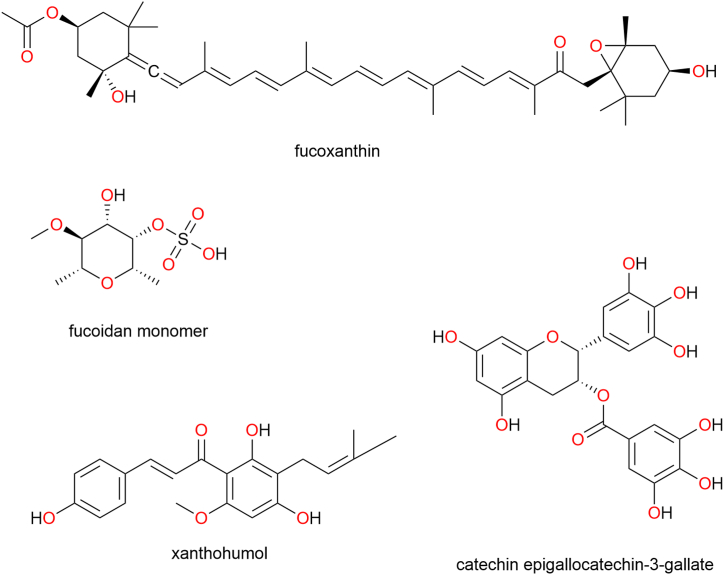
Natural compounds to treat heart-related diseases and their chemical structures.

Along with diabetes, cancer, and chronic respiratory illness, cardiovascular disorders are one of the four main non-communicable diseases (NCDs) accounting for serious concerns [32,89,177]. According to the World Health Organization, cardiovascular illnesses were responsible for 17.9 million deaths in 2016, or 44% of all NCD deaths [135, 295]. Thus, CDV constitute the main cause of death around the globe [295]. CDV are currently among the leading causes of death around the world [268]. Current CVD disease treatment strategies make use of a wide range of potent pharmaceutical options. Unfortunately, most of these medications have a poor safety record and cause severe adverse effects [276]. In the search for new drug leads, natural products have long been held in high regard. The potential of several natural products as sources of treatments for cardiovascular diseases is increasingly being recognized [242]. Natural products can contribute numerous advantages to treatment plans via a wide variety of processes. The first step in delaying the beginning and progression of coronary artery disease (CAD) is to prevent the oxidation of LDL cholesterol [257,312], which may be accomplished with the use of products with antioxidant activity. Also, in patients with advanced CAD, antioxidant medications protect against oxidative damage brought on by ischemia/reperfusion [297, 298]. In addition, they boost nitric oxide levels, which benefits cardiovascular and endothelial function [165]. Second, their anti-inflammatory properties aid in protecting against reperfusion injury, atherosclerotic, myocardium hypertrophy, and vascular plaque development [265,278]. Third, the plasma lipids profile can be improved by using some natural products, and these products have powerful anti-atherogenic actions like in resveratrol [192, 213, 215]. It is possible that natural product has curative effects beyond just antioxidant and anti-inflammatory ones, including anti-apoptotic [191], anticoagulant [271], vasodilatory [259], and diuretic [149]. Therefore, numerous natural products have the clinical potential to treat heart-related diseases, as shown in Table 7 and Fig. 7.

**Table 7:** Clinical trials of natural products to treat heart-related diseases (https://clinicaltrials.gov).

Compound	ClinicalTrials.gov Identifier	Type of study	Characteristics of patients (n)	Dose and time of treatment	Condition	Phase	Additional Refs
Fucoxanthin and oligo fucoidan	NCT02875392	randomized, Interventional, placebo-controlled trial	FuciHiQ group (n = 21) or placebo group (n = 21)	FucoHiQ (275 mg Oligo Fucoidan + 275 mg HS Fucoxanthin) 550mg/capsule 6 per day	non-alcoholic Fatty Liver Disease	–	[100]
Xanthohumol	NCT01367431	Observational	20 mg group, 60 mg group and 180 mg group; n = 48	one capsule of one of the three doses (20, 60, 180 mg) randomly assigned	heart disease	–	
Cocoa polyphenols	NCT00654862	randomized, Interventional, placebo-controlled trial	250 mg group, 1000 mg group, placebo; n = 48	oral administration of capsules with 1000 or 250 mg polyphenols	hypertension	phase 1	
Catechin epigallocatechin-3-gallate (EGCG)	NCT01662232	randomized, Interventional, placebo-controlled trial	200 mg group, placebo group; n = 50	200 mg EGCG	cardiovascular diseases	–	
Exenatide	NCT00650546	Open label	N = 8	5 mcg twice a day titrated to 10 mcg twice a day	nonalcoholic fatty liver disease	phase 2/3

#### 1.4.4 Natural products with anticancer activity

**Fig. 8:**
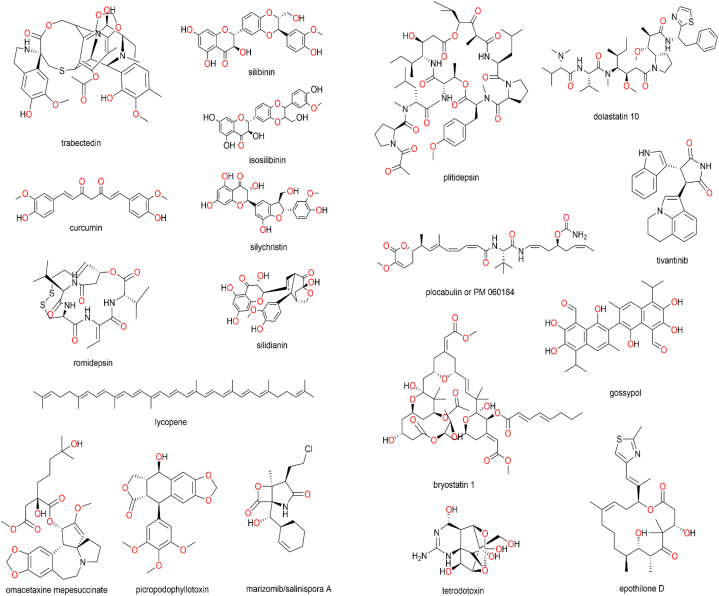
Natural compounds with anti-cancer activity and their chemical structures.

Global cancer registry expansion has stimulated research into potential new treatments that are selectively toxic to cancer cells while being safe for healthy tissue [4]. Previous anticancer medications showed relatively high toxicity not only to the tumor cells, but also to the normal cells of the body portion where the cancer had formed [207]. New anticancer medications are currently being researched both from various sources including marine and terrestrial [50, 71]. Medical practitioners have relied on plants for ages to treat a wide variety of conditions. Some plants are used for their medicinal properties and consumed as part of local folk medicine in many different cultures. As the number of people diagnosed with cancer rises, including breast cancer [290], so does the demand for effective treatments. After being extracted and purified, many different plant-based anticancer drugs are tested on cells (including various cancer cell lines) and experimental animals. The discovery of significant biological activity in many plants with a history of use in traditional medicine has led to their inclusion into mainstream medicine [224]. These compounds can be obtained, for example, through plant extracts. Alternatively, combination of biology, chemistry, and technologies can be used to synthesize plant-based anticancer compounds [251]. There are several kinds of chemicals found in nature (including plants and aquatic creatures) that display anticancer effects, such as diterpenes, quinone, peptides and their cyclic form, alkaloids, purine, sesquiterpene, and macrocyclic polyether. It is generally more cost-effective to obtain these substances from their natural sources than to prepare them synthetically. Moreover, numerous natural products have the clinical potential to cancer, as shown in Table 8 and Fig. 8.

**Table 8:** Clinical trials of natural products to as anti-cancer agents (https://clinicaltrials.gov).

Compound	ClinicalTrials.gov Identifier	Type of study	Characteristics of patients (n)	Dose and time of treatment	Condition	Phase	Additional Refs
Trabectedin	NCT01343277	A multicenter, open- label, randomized, active- controlled, parallel- group	trabectedin group (n = 378) or dacarbazine group (n = 172)	trabectedin Arm: 1.5 mg/m^2^ as a 24 h IV infusion q3wk.	advanced liposarcoma, Leiomyosarcoma	phase 3	[100]
Sylmarin (mixture of flavonolignans consisting of silibinin, isosilibinin, silychristin, silidianin)	NCT03130634	open-label, randomized, comparative, double arm, single center	sylmarin group or control group; n = 70	during six cycles of FOLFIRI chemotherapy, the patients will take silymarin (150 mg) 3x daily from day 1 to day 7 during one cycle of treatment.	Metastatic, colorectal cancer	phase 4	
Silibin-Phytosome	NCT00487721	non-Randomized	silibin-phytosome group or control group; n = 12	13 g daily, in three divided doses for 2–10 weeks.	prostate cancer	phase 2	
Xanthohumol	NCT02432651	randomized	2 mg group, 12 mg group and 24 mg group, placebo; n = 64	2/12/24 mg xanthohumol at breakfast, lunch, and dinner for 3 weeks.	oxidative Stress	Phase 1	
Catechin (Sinecatechins 10%)	NCT02029352	randomized double-blinded	catechin group or placebo group; n = 42	twice daily (morning and evening) in a thin layer to the tumor including 5 mm of the surrounding skin	carcinoma	Phase 2/3	
Lycopene	NCT00068731	randomized double-blinded	lycopene group or placebo group; n = 47	twice daily on days 1–28. Courses repeat every 28 days for at least 4 months	prostate cancer	phase 2	
Catechin epigallocatechin-3-gallate (EGCG)	NCT02577393	randomized double-blinded	prophylactic EGCG group, therapeutic EGCG group, placebo; n = 83	440 lmol/L	lung neoplasms	phase 2	
Curcumin	NCT01740323	randomized double-blinded	resveratrol group (n = 15) or placebo group (n = 15)	500 mg BID	breast cancer	phase 2	
Resveratrol	NCT00256334	randomized, placebo- controlled, double blind	resveratrol group, placebo group; n = 11	one of four dose cohorts: plant-derived resveratrol tablets at a dose of 80 mg/day, plant-derived resveratrol tablets at a dose of 20 mg/day, Grape Powder (GP) at a dose of 120 g/day, and GP at a dose of 80 g/day.	colon cancer	phase 1	
Resveratrol	NCT00920803	double-blind, randomized	resveratrol group, placebo group; n = 9	5 g once daily for 14 days	neoplasms, colorectal	phase 1	
Resveratrol	NCT00433576	non-Randomized	n = 20	STAGE II: Patients receive oral resveratrol on days 1–8. Patients undergo colorectomy on day 9	aAdenocarcinoma of the Colon Adenocarcinoma of the Rectum Stage I Colon Cancer Stage I Rectal Cancer Stage II Colon Cancer Stage II Rectal Cancer Stage III Colon Cancer Stage III Rectal Cancer	phase 1	
Sulforaphane	NCT00982319	randomized double-blinded	n = 34	100 μmols of sulforaphane dissolved in 150 mL mango juice once a day for 14 days	breast cancer	phase 2	
Romidepsin	NCT00106418	non-randomized, multicenter, open-label trial	n = 35	13 mg/m^2 of romidepsin intravenously over 4 h on Days 1, 8, and 15 of each 28-day cycle	prostate cancer	phase 2	
Romidepsin	NCT01353664	open-label, single-arm study	n = 19	same dose, infusion time and frequency used for the last dose of romidepsin given	lung cancer	phase 2	
Omacetaxine mepesuccinate	NCT00375219	open-label	chronic phase group (n = 62), accelerated phase (n = 20), blast phase (n = 21)	1.25 mg/m^2 subcutaneously, twice daily for 14 consecutive days every 28 days until response	chronic myeloid leukemia	phase 2	
Picropodophyllotoxin	NCT01466647	open single-center, explorative	n = 12	a repeated BID treatment for 14 days, followed by a 7-day observation period for two treatment periods	non-small Cell Lung Cancer	phase 1	
Picropodophyllotoxin	NCT01561456	open label, randomized, multi-center	n = 100	oral suspension at 400 mg twice daily for 21 days per cycle	non-small-cell Lung Cancer Squamous Cell Carcinoma Adenocarcinoma of the Lung	phase 2	
Marizomib/salinosporamide A	NCT00396864	multicenter, open-label study	n = 51	injection at doses ranging from 0.0125 to 0.8 mg/m2 over 1–10 min on Day 1, Day 8, Day 15 of each 28-day Cycle; 11 dose cohorts during dose-escalation	cancer lymphomas	phase 1	
Plitidepsin	NCT00229203	non-randomized, multicentre, open-label	plitidepsin group (n = 32) and plitidepsin with dexamethasone (n = 19)	5 mg/m2, 3-h infusion every 2 weeks	Multiple Myeloma	phase 2	
Plitidepsin	NCT01102426	non-randomized, multicentre, open-label	plitidepsin+ dexamethasone Group (n = 171), dexamethasone (n = 84)	5 mg/m2 intravenously (i.v.) over 3 h on Day 1 and 15 every 4 weeks. dexamethasone: 4 mg tablet. 40 mg orally on Day 1, 8, 15 and 22 every four weeks at least 1 h before plitidepsin infusion.	multiple myeloma	phase 3	
Plocabulin/PM 060184	NCT03427268	open-label, multicentre study	PM 060184 group (n = 32)	9.3 mg/m2 PM 060184 i.v. as a 30-min infusion via a central or peripheral venous catheter; It administered on Day 1 and Day 8 q3wk	colorectal cancer	phase 2	
Bryostatin 1	NCT00003968	open Label	n = 35	bryostatin 1 IV over 1 h on days 1, 8, and 15. Treatment continues every 4 weeks in the absence of unacceptable toxicity or disease progresssion.	kidney cancer	phase 2	
Tetrodotoxin	NCT00725114	multicentre, Randomized, Double-blind, Placebo-controlled, Parallel-design	tetrodotoxin group, placebo group; n = 165	30 μg twice daily for 4 days	cancer pain	phase 3	
Tivantinib	NCT01755767	randomized, double-blind study	tivantinib 240 mg BID Cohort group (n = 28), Placebo Matching 240 mg BID Cohort group (n = 15), Tivantinib 120 mg BID Cohort group (n = 226), Placebo Matching 120 mg BID Cohort group (n = 114)	the dosage of 120/240 mg tablets administered by mouth twice daily (BID), once in the morning and once in the evening, with food, for a total daily dose of 240/480 mg.	hepatocellular carcinoma	phase 3	
Tivantinib	NCT02029157	randomized double-blind, placebo-controlled	tivantinib 120 mg BID Cohort group (n = 134), Placebo Matching 120 mg BID Cohort group (n = 61)	twice-a-day oral tivantinib (120 mg bid)	liver cancer	phase 3	[146]
Gossypol	NCT00540722	Open-label	gossypol group (n = 56)	once daily on days 1–21. Treatment repeats every 28 days	glioblastoma	phase 2	
Epothilone D	NCT00077259	open-label	n = 16–69	drug IV over 90 min on days 1, 8, and 15. Courses repeat every 28 days	colorectal cancer	phase 2	
Dolastatin 10	NCT00003677	open-label	n = 9	IV bolus once every 21 days.	pancreatic cancer	phase 2

### 1.5 Drug delivery approaches for natural products in the management of metabolic disorders

Recently, the use of natural products as the main active agents for the treatment of numerous diseases, including metabolic disorders. Due to the limitation of bioactive compounds the natural products, several drug delivery approaches have been developed to overcome the problems [125, 197, 264, 280]. In this review, we showed numerous approaches containing natural compounds to the treatment of metabolic disorders.

#### 1.5.1 Drug delivery approaches for natural products in the management of diabetes

Grape (*Vitis vinifera*) has been well-known to possess phenolic compounds, showing antioxidant activities. Gharib and coworkers investigated two major phenolic compounds in the grape, cyanidin and delphinidin as antidiabetic agents in the form of liposomal delivery system [84]. Liposomal system has been widely used to improve the efficacy of many drugs to treat some diseases [35, 94, 116, 133, 221, 302]. In their study, two compounds were incorporated into liposomes using an extrusion technique, showing the entrapment efficiencies of more than 80% for both compounds. It was found that free drugs could reduce the glycation of albumin in vitro study to 30.5% for delphinidin and 46% for cyanidin. Interestingly, following the formulation into liposomal system, the glycation of albumin values was 8.5% for delphinidin and 14.6% for cyanidin. Furthermore, *in vivo* study showed that the liposomal could exhibit higher anti-glycation efficacy compared to free compounds. In the diabetic mice, the administration of liposomal was able to significantly reduce the albumin and HbA1c glycation rate in comparison to free compounds. Accordingly, this showed the feasibility of the formulation of natural compounds in the improvement of diabetic therapy.

In another study, using similar system, Yücel and co-workers encapsulated a natural compound, resveratrol into two different types of liposomes, PEGylated and non-PEGylated [307]. In their study, the combination of dipalmitoylphosphatidylcholine (DPPC) and cholesterol was used to prepare multi-bilayered particles with size of 215 nm. The diabetic (streptozotocin-induced) pancreatic cell line was treated with resveratrol solution and resveratrol-loaded liposomes for 24 h. The findings showed that insulin concentrations increased, with a greater degree in the liposome formulations treated groups, whereas glucose concentrations decreased. In vitro study, it was found that liposomal formulation could show a significant antioxidant activity in pancreatic cells compared to free solution. Thus, this could show the promising approach in the therapy of diabetes mellitus and associated oxidative stress.

Mao et al. explored the benefit of *Echinacea purpurea* as antidiabetic agent [171]. It has been reported that the extract of *E. purpurea* contains numerous phenolic compounds and isobutylamides, exhibiting antidiabetic activity. To further improve the effectiveness, the extract was incorporated into chitosan/silica nanoparticles with particle size of 218 nm, 66.9% of entrapment efficacy and 39.9% of drug loading. Furthermore, it was found that the formulation could reduce the oxidative stress in LC-540 cells with strong antioxidant activity. Importantly, in the *in vivo* study using diabetes rat models induced by streptozotocin (STZ), the nanoparticles could reduce the glucose blood level to the normal rate, increase the resistance of insulin and the resistance of plasma fibroblast growth factor 21 (FGF 21), compared to the free form.

Another type of nanoparticles, gold nanoparticles were also investigated for their antidiabetic activity. Daisy and team synthetized gold nanoparticles using *Cassia fistula* stem bark aqueous extract [52]. Gold nanoparticles have been greenly synthetized using many natural compounds [119, 136, 181, 188, 208, 237, 241]. In this study, numerous characterizations were carried out, including ultraviolet–visible spectroscopy, Fourier transform infrared spectroscopy, and scanning electron microscopy to investigate their absorbance pattern, the possible functional groups, the size of the nanoparticles, respectively. Overall, the results showed that the gold nanoparticles prepared from *C. fistula* stem bark aqueous extract exhibited promising hypoglycemic activity compared to aqueous extract according to the analysis of level of serum glucose, body weight, kidney function evaluation, liver function evaluation, and profile of lipid. It was found that the administration of gold nanoparticles could decrease serum biochemistry parameters in rats with streptozotocin-induced diabetes. Therefore, this showed the potency of gold nanoparticles of *C. fistula* to improve the diabetic therapy.

#### 1.5.2 Drug delivery approaches for natural products in the management of obesity

The application of drug delivery system containing natural compounds has been also used in the treatment of obesity. One of Ayurvedic medicine, *Salacia chinensis*, has been reported to show potential pharmacological effects. Gao and team developed gold nanoparticles loading *S. chinensis* to investigate its anti-obesity activity [83]. The study was conducted in a high-fat diet (HFD) treated obese rats. Initially, the nanoparticles prepared were characterized for their physicochemical parameters. The results showed that the formulation exhibited a spherical shape with crystal form. Essentially, in the *in vivo* study, the nanoparticles could reduce several obesity parameters in the HFD rats, including the bodyweight changes, resistin, adipose index, inflammatory markers, BMI, leptin, CRI, adiponectin, AI, liver marker enzymes, lipid profile, dan AMPK signaling proteins. Furthermore, the liver histopathological evaluation showed a promising result with the reduction of hepatocyte degradation following the administration of nanoparticles of Salacia chinensis. Using similar approach, Ansari et al. developed gold nanoparticles synthesized using *Smilax glabra* rhizome [13]. The nanoparticles were 21 nm in size with excellent cell uptake property. It was found that the administration of the nanoparticle in HFD rats showed superior antiobesity activity based on several parameters, including lipid profile, liver markers, hormones like leptin, adiponectin and resistin, as well as histopathological evaluations.

To overcome the bioavailability and solubility of issue of resveratrol as antiobesity agent, Wan and coworkers formulated PLGA nanoparticles loading resveratrol [281]. The nanoparticles were prepared using oil in water emulsion method, producing particles with size of 176.1 nm and zeta potential of −22.6 mV. Moreover, the entrapment efficiency and the drug loading were found to be 97% and 14.9%, respectively with sustained release behavior in the gastrointestinal tract and excellent physical stability profiles. Importantly, compared to free resveratrol, the administration of PLGA nanoparticles showed a better antiobesity activity through lipogenesis, enhancing lipolysis and lowering hepatocellular proliferation. Morover, Andelbaky et al. isolated cellulose nanocrystal from grape and investigated the antiobesity activity [12]. The nanocrystal was isolated using sodium hydroxide and bleached using sulphuric acid. In the rat obesity model, by observing the body weight, the lipid profiles, liver function and kidney function, the nanocellulose showed antiobesity activity compared to the positive control grape seed powder.

Using different administration route, Ariamoghaddam et al. developed nanofibers patches for transdermal delivery of curcumin [15]. Transdermal route has been used to deliver numerous drugs as alternative to the conventional oral route. Several studies have shown that the administration of bioactive compounds via this route could result in better bioavailability compared to other routes [35, 67, 133, 190, [194], [195], [196], 221, 263, 279]. The nanofibers were fabricated using polyvinyl alcohol and gelation, producing formulation with fiber diameter of 200–250 nm and highly reproducible. The effectiveness of transdermal delivery was evaluated by observing the body weight, the level of blood parameters and MRI imaging. It was found that the level of leptin decreased following the transdermal delivery of curcumin using this approach. Importantly, MRI imaging showed the decrease of adipose tissue around 4–7%. Accordingly, this showed that the transdermal delivery could be an alternative delivery route of natural compounds for obesity therapy.

#### 1.5.3 Drug delivery approaches for natural products in the management of heart related diseases

With respect to the application of drug delivery system of natural product in the treatment of heart related diseases, polyphenol has been still widely used. For example, Qi et al. developed self-assembly nanoparticles from several types of polyphenol, namely gallic acid, catechin, tannic acid and epigallocatechin gallate, to prepare functionalized nanoparticles [210]. The four polyphenols have different type of phenolic hydroxyl groups and following optimization process, combined with cyclodextrin, the use of tannic acid to prepare the nanoparticles showed the optimum formulation with potent antioxidant activity. The results showed that the nanoparticles could potentially protect the cells from hypoxic-ischemic injury. In vivo study, following intravenous injection in the ventricular fibrillation cardiac arrest model in rats and myocardial hypertrophy model in mice, the formulation localized in the injured heart. In the two models, the nanoparticles were able to result in significant pharmacological effects. Therefore, this could be a promising system for the treatment of heart-targeting diseases.

Another study highlighted the formulation of zinc oxide nanoparticles containing *Artemisia herba-alba* leaves’ extract (AHALE) to improve the cardioprotective effect of AHALE [8]. The efficacy study was carried out in myocardial infarction model in male rats induced by isoproterenol. Several parameters were investigated, showing that the administration of the nanoparticles could increase the level of heart markers, lipid profile markers and lipid peroxidation products compared to free AHALE. Moreover, the reduction of the activity of antioxidant activity was found in the animal model following the administration of this approach. In addition, they also investigated the effect of the administration of the nanoparticles before the inducement of isoproterenol and they found that the oxidative stress could be avoided. Therefore, this system could also be used to prevent the heart diseases. With the same purpose, the development of silver nanoparticles from *Mentha piperita*, stabilized by chitosan was conducted by Wang and team [286]. Silver nanoparticles have been found to show numerous pharmacological effects [181, 193, 286]. In this study, the nanoparticles were found to possess sizes around 5 nm–15 nm with spherical shape. The formulation was administered orally in rats with heart failure model, and it was found that the size of the infarct was significantly reduced and the function of the cardiac was improved, indicated by lower left ventricular end diastolic pressure and raised ± dp/dt(max).

Furthermore, Tan et al. encapsulated total flavonoid extract from*Dracocephalum moldavica* L. (TFDM) with myocardial protective activity in solid lipid nanoparticles [258]. This study was designed due to the low solubility of the flavonoid compounds in the extract. The nanoparticles were optimized using central composite design, resulting in optimum formulation with size of 104.83 nm, PDI value of 0,201 and zeta potential of −28.7 mV. Importantly, the *in vivo* studies showed significant higher myocardial protection compared to free extract, according to the area of infarct, histopathological evaluation, cardiac enzyme parameters and serum inflammatory factors.

In terms of another type of heart related disease, Yu and team developed smart delivery containing polyphenol compounds for thrombolytic therapy [305]. The system consisted of thrombin-responsive nanoparticles prepared via noncovalent interactions form tannic acid to cross-link urokinase-type PA (uPA) and a thrombin-cleavable peptide on a sacrificial mesoporous silica template. The results showed that the nanoparticles could hold active uPA. Importantly, in the presence of thrombin, the nanoparticles showed improved the activation of plasminogen, indicating the responsive behavior of the system.

#### 1.5.4 Drug delivery approaches for natural products in the management of cancer

Natural products have long been utilized as medications with pharmacological actives to aid in the treatment of a wide range of medical conditions. Even so, our understanding of their potential as materials remains limited. Natural compounds of small molecular weight extracted from traditional Chinese medicine have been demonstrated to exhibit novel properties in recent years, including the ability to self-assemble into gels (i.e., natural product gels, NPG). However, there is a lack of competence in the application development of these natural compounds, which significantly reduces their practical worth and slows the improvement of natural products in industrial area. Therefore, Zhi et al. used a family of triterpenoid natural compounds with its own ability to self-assemble (gel scaffolds material) for the development of drug delivery systems. Remarkably, these NPG were not only enabled synergistic treatment of cancers via bioactive natural products, but also displayed remarkable self-healing, regulated gelation, good safety, and prolonged release. When it comes to tumor therapy, NPG scaffolds have many advantages than non-bioactive gel scaffolds. These include more tumor inhibition, improved health and body recovery, enhanced immune system, fewer toxic side effects, and increased chances of survival. Constructing NPG scaffolds is a significant step toward the discovery of novel uses for natural products, as it makes full use of these materials in their self-assembled form [320].

Chemoprevention of associated-colorectal cancer (CRC) in patients with inflammatory bowel disease (IBD) has shifted to prioritize anti-inflammatory therapies. Current anti-inflammatory medications used in IBD therapy have not yet been studied enough for their potential chemopreventive effects. For this reason, research exploring novel chemopreventive possibilities is essential, and natural compounds derived from food and complementary and alternative medicine have become attractive resources owing to their multi-component nature and ability to target multiple cancer types. Danggui Decoction (DGD) is a traditional Chinese medicinal formula for the treatment of inflammatory bowel disease (IBD) that includes the ingredients *Angelicae sinensis* Radix, Zingiberis Rhizoma Recens, and Jujubae Fructus; DGD supercritical fluid extracts (DGDSFE) and DGD polysaccharide extracts (DGDPE) are promising candidates for chemoprevention treatment. To explore this promising activity, Liu and coworkers used extrusion-spheronization and coating technologies to create a multi-unit pellet drug delivery system (MUPDDS) with two separate components: pellets containing DGDSFE for colon targeting and pellets containing DGDPE for peripheral targeting [159]. This MUPDDS was tested for its chemopreventive properties in a rat model of cancer which formerly induced with 1,2-dimethylhydrazine and sodium dextran sulfate. Serum levels of TNF-α, IL-1β, and hepcidin were reduced, while levels of IFN-γ and IL-2 in splenocyte supernatant were elevated, indicating anti-inflammation, iron metabolism regulation, and immune regulation of DGDSFE and DGDPE in MUPDDS, which led to a decrease in tumor incidence, tumor number, and tumor volume after 14 weeks of daily administration. In addition, a comparison with extracts, DGDSFE colon-targeted pellets, and DGDPE pellets showed the feasibility and advantage of MUPDDS in chemoprevention, presenting an encouraging technique to improve the effect of traditional Chinese medicines in cancer prevention.

The use drug delivery containing natural products has also been applied for the treatment of hepatocellular cancer. Patients with unresectable metastatic or recurrent hepatocellular cancer continue to benefit most from the use of combination chemotherapy medication. It is well known that there is also a significant advance in the management of this disease. It has been found that the immunomodulator called lentinan, which has been used in the treatment of cancer, also possesses anti-tumor activities. Lentinan has been shown to inhibit hepatocellular cancer, though the exact processes by which this occurs are not yet understood. *In vitro* and *in vivo* studies using HepG2 cells and H22 tumor-bearing mice demonstrated that Lentinan strongly synergizes with oxaliplatin in inhibiting NF-κB, Stat3, and survivin signaling via the mitochondrial route. Additionally, Lentinan reduced oxaliplatin's negative effects. In light of these results, Lentinan was proposed as a promising drug for use in combination with oxaliplatin in the treatment of hepatocellular carcinoma [315].

Many bioactive substances are now collected from nature, particularly those that have anticancer effects. As anticancer, these substances can alter the signaling pathways involved in the cell cycle, decrease interactions between cytoskeleton components, or overexpress antitumoral proteins. To increase pharmacokinetic and pharmacodynamic parameters, these drugs' physicochemical characteristics and targeted delivery effectiveness may be modified. The delivery method of exosomes, which is enhanced by a number of features, has the potential to make them the next generation of transporters for therapeutic compounds. Exosomes are a subtype of cellular vesicles (30–150 nm) derived from membranes that are crucial for intercellular communication. Numerous uses, including medicine delivery, have been developed for these nanovesicles due to their inherent capacity as nanocarriers [2, 91, 123, 154, 250]. Donoso-Quezada and coworkers showed that plant-derived bioactive substances, like saponins and flavonoids from black bean extract, may be integrated into the exosomal structure and taken up by recipient cells *in vitro*. According to our preliminary research, exosomal formulations of the extract appear to increase the antiproliferative response, adding to our understanding of the characteristics of exosomes as nanocarriers. In the short term, our effort will be focused on extending the *in vitro* data supporting the increased activity of exosomal formulations, and in the medium term, we will work on the creation of new, more effective methods to create, isolate, and purify exosomes [63].

In order to increase the response *in vitro*, in another study, they loaded exosomes isolated from various cell lines with saponins and flavonoids from a black bean extract (*Phaseolus vulgaris* L.) with antiproliferative activity. In order to transfer these chemicals to recipient cells, they demonstrated that exosomes might be loaded with at least three different phytochemicals in a single step. Additionally, they discovered that the exosomal extract has higher bioactivity than that of other formulations of the same extract. Exosomes offer a possible alternative, according to our findings, for enhancing the delivery of complex combinations of bioactive chemicals, such as plant extracts. Therefore, developing novel products for human use with improved nutraceutical characteristics may be one of the future uses for these nanovesicles [64].

Another type of approach used for the cancer treatment is mesoporous silica nanoparticles. Mesoporous silica nanocarriers for drug delivery were developed by Porrang and team from natural materials, including rice and wheat husk [203]. By using acid leaching, the biogenic silica in grain husk was first removed, and it was subsequently transformed into sodium silicate as a silica precursor. Subsequently, using continuous and discrete sol-gel methods, sodium silicate was added to the template mixture to create mesoporous silica nanoparticles. The XRD, FT-IR, BET, and SEM analyses were used to examine the impacts of natural source type and precursor addition method on the morphological and physicochemical features of nanocarriers. Their findings indicated that spherical nanocarriers made of rice husk were more crystalline and had slit-shaped pores. The results also demonstrated that the discrete addition of the precursor improved their hydrophilicity, particle size, and pore size in contrast to continuous addition, most likely as a result of the precursor's high starting concentration in the reaction mixture. Model anticancer drug doxorubicin (DOX) was loaded into the nanocarriers, and the behavior of the drug release was examined at two different pH values (7.4 and 5.4). Due to DOX increased solubility in an acidic environment, the accumulated released drug at pH 5.4 was generally around twice as much as pH 7.4. Additionally, due to their larger pore diameters than continuous mode nanocarriers, discrete mode nanocarriers had higher cumulative released drug concentrations at pH 5.4. On the HFF-2 and MCF-7 cell lines, respectively, the biocompatibility and cytotoxicity of nanocarriers and nanocarriers loaded with DOX were also examined. Additionally, a morphological analysis of the MCF-7 cells was used to assess apoptosis as the mechanism of cell death. The DOX-loaded nanocarriers, particularly discrete mode produced nanocarriers, displayed high-efficiency anticancer action on the MCF-7 cell line within tolerable toxicity limits and apoptosis induction.

## 2 Concluding remarks and future perspectives

Over the last two decades, tremendous efforts have been made to reveal the mechanistic basis of metabolic disorders and prospective biological targets that are clinically translatable and important in the pharmacological management of the diseases. From numerous experimental findings, we now know that metabolism and immunity are interconnected and serious malfunction in the regulatory networks can result in the development of inflammation-induced metabolic diseases, including diabetes, obesity, cardiovascular diseases, and cancer. Hence, the interconnected biological interface of immune system and metabolism has been suggested to play a tremendous role in the homeostatic mechanism to maintain humans’ health. Nevertheless, despite advancement in the diagnostic tools and clinical procedures to detect the hallmarks of metabolic disorders, and rapid progress in the discovery and development of safe and potent drugs to treat metabolic disorders, the number of FDA-approved drugs to manage metabolic disorders remains low. To improve this number, efforts to utilize natural products and their isolated compounds are expanding.

Metabolic disorders are multifactorial; thus, the use of multiple medications may be required to achieve a proper pharmacological response. To this end, a less-risky use of pharmaceutical preparations, such as natural products, may play a beneficial role. Indeed, it has been widely suggested that natural products have a low risk to elicit dangerous adverse effects and such feature can be safely used in the urge to treat metabolic disorders. However, it is important to note that slow pace of animal and clinical studies to demonstrate the efficacy and safety of natural products has been one of the most unsettling avenues in the scientific efforts to advance biomedical and pharmaceutical research in this field. Therefore, it is crucial to tackle this problem as fast and as decisive as possible to minimize the gap in providing scientific evidence for the benefits of plant-derived phytochemicals in the management of metabolic disorders. Such endeavor shall provide valuable support in the long-term battle against the increasing incidence of metabolic disorders-related diseases.

## Data availability statement

Data will be made available on request.

## Author contributions

F.N. and J.S-G. designed the outline of the manuscript, F.N., A.F., S.S.M., A.D.P., M.S., and D. C wrote the initial draft, F.N., A.F., S.S.M., A.D.P., M.S., D.C., T.B.E., and J.S-G revised the manuscript critically for important intellectual content. All authors contributed to the article and approved the submitted version.

## Declaration of competing interest

The authors declare that they have no known competing financial interests or personal relationships that could have appeared to influence the work reported in this paper.
